# Impact of N-Alkylamino Substituents on Serotonin Receptor (5-HTR) Affinity and Phosphodiesterase 10A (PDE10A) Inhibition of Isoindole-1,3-dione Derivatives

**DOI:** 10.3390/molecules25173868

**Published:** 2020-08-25

**Authors:** Anna Czopek, Anna Partyka, Adam Bucki, Maciej Pawłowski, Marcin Kołaczkowski, Agata Siwek, Monika Głuch-Lutwin, Paulina Koczurkiewicz, Elżbieta Pękala, Anna Jaromin, Bożena Tyliszczak, Anna Wesołowska, Agnieszka Zagórska

**Affiliations:** 1 Department of Medicinal Chemistry, Jagiellonian University Medical College, 9 Medyczna Street, 30-688 Krakow, Poland; adam.bucki@uj.edu.pl (A.B.); maciej.pawlowski@uj.edu.pl (M.P.); marcin.kolaczkowski@uj.edu.pl (M.K.); agnieszka.zagorska@uj.edu.pl (A.Z.); 2 Department of Clinical Pharmacy, Jagiellonian University Medical College, 9 Medyczna Street, 30-688 Krakow, Poland; annairena.partyka@uj.edu.pl (A.P.); a.wesolowska@uj.edu.pl (A.W.); 3 Department of Pharmacobiology, Jagiellonian University Collegium Medicum, 9 Medyczna Street, 30-688 Krakow, Poland; agat.siwek@uj.edu.pl (A.S.); monika.gluch-lutwin@uj.edu.pl (M.G.-L.); 4 Department of Pharmaceutical Biochemistry, Jagiellonian University Collegium Medicum, 9 Medyczna Street, 30-688 Krakow, Poland; paulina.koczurkiewicz@uj.edu.pl (P.K.); elzbieta.pekala@uj.edu.pl (E.P.); 5 Department of Lipids and Liposomes, Faculty of Biotechnology, University of Wroclaw, 14a Joliot-Curie, 50-383 Wroclaw, Poland; anna.jaromin@uwr.edu.pl; 6 Faculty of Materials Engineering and Physics, Cracow University of Technology, Institute of Materials Science, 24 Warszawska Street, 31-155 Krakow, Poland; bozena.tyliszczak@pk.edu.pl

**Keywords:** benzimidazole derivatives, phosphodiesterase 10A, schizophrenia, antipsychotic activity

## Abstract

In this study, a series of compounds derived from 4-methoxy-1*H*-isoindole-1,3(2*H*)-dione, potential ligands of phosphodiesterase 10A and serotonin receptors, were investigated as potential antipsychotics. A library of 4-methoxy-1*H*-isoindole-1,3(2*H*)-dione derivatives with various amine moieties was synthesized and examined for their phosphodiesterase 10A (PDE10A)-inhibiting properties and their 5-HT_1A_ and 5-HT_7_ receptor affinities. Based on in vitro studies, the most potent compound, **18** (2-[4-(1*H*-benzimidazol-2-yl)butyl]-4-methoxy-1*H*-isoindole-1,3(2*H*)-dione), was selected and its safety in vitro was evaluated. In order to explain the binding mode of compound **18** in the active site of the PDE10A enzyme and describe the molecular interactions responsible for its inhibition, computer-aided docking studies were performed. The potential antipsychotic properties of compound **18** in a behavioral model of schizophrenia were also investigated.

## 1. Introduction

**Figure 1:**
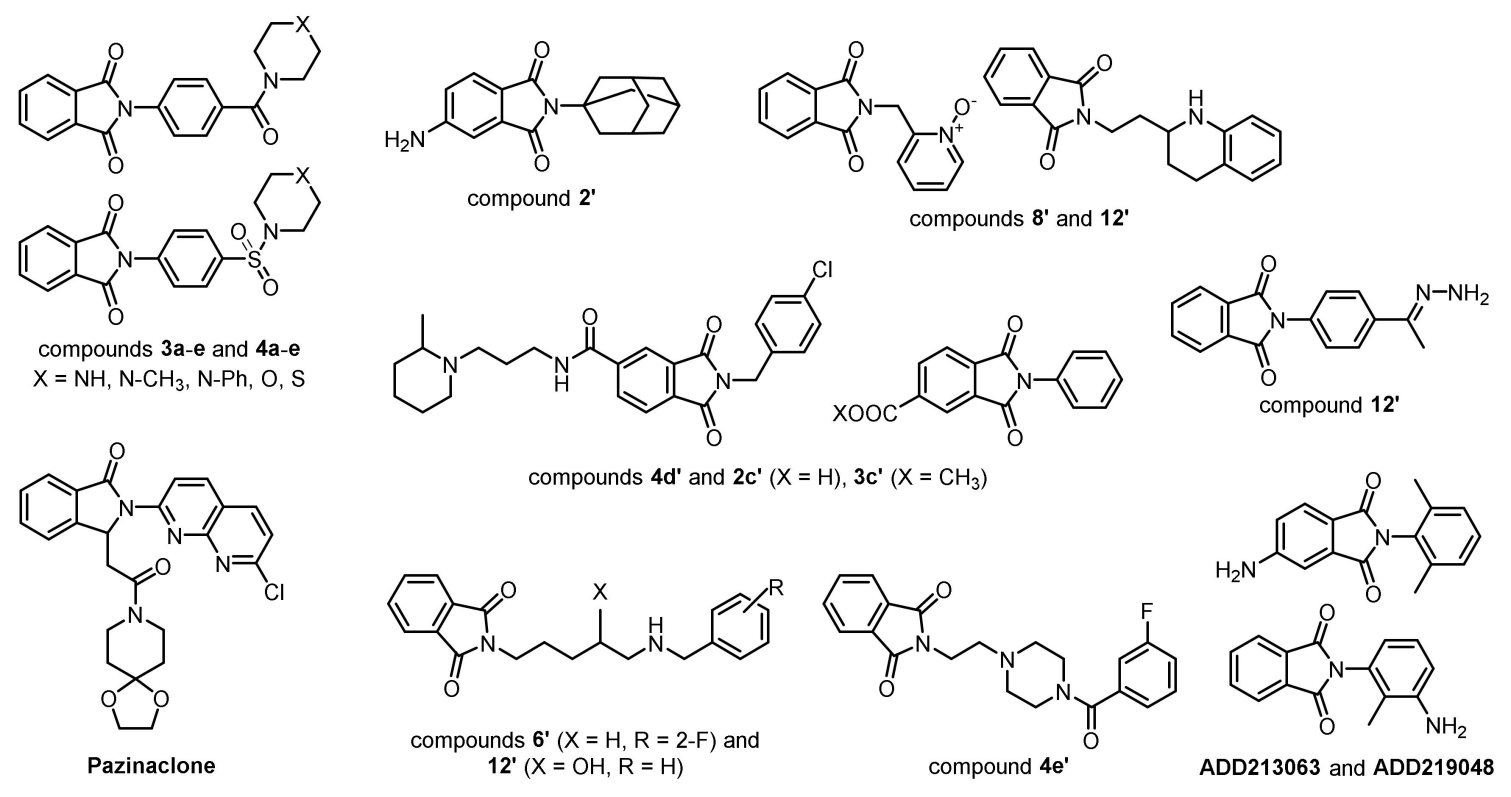
Examples of phthalimide derivatives with various biological activity [1,2,3,4,5,6,7,8,9,10,11,12].

**Figure 2:**
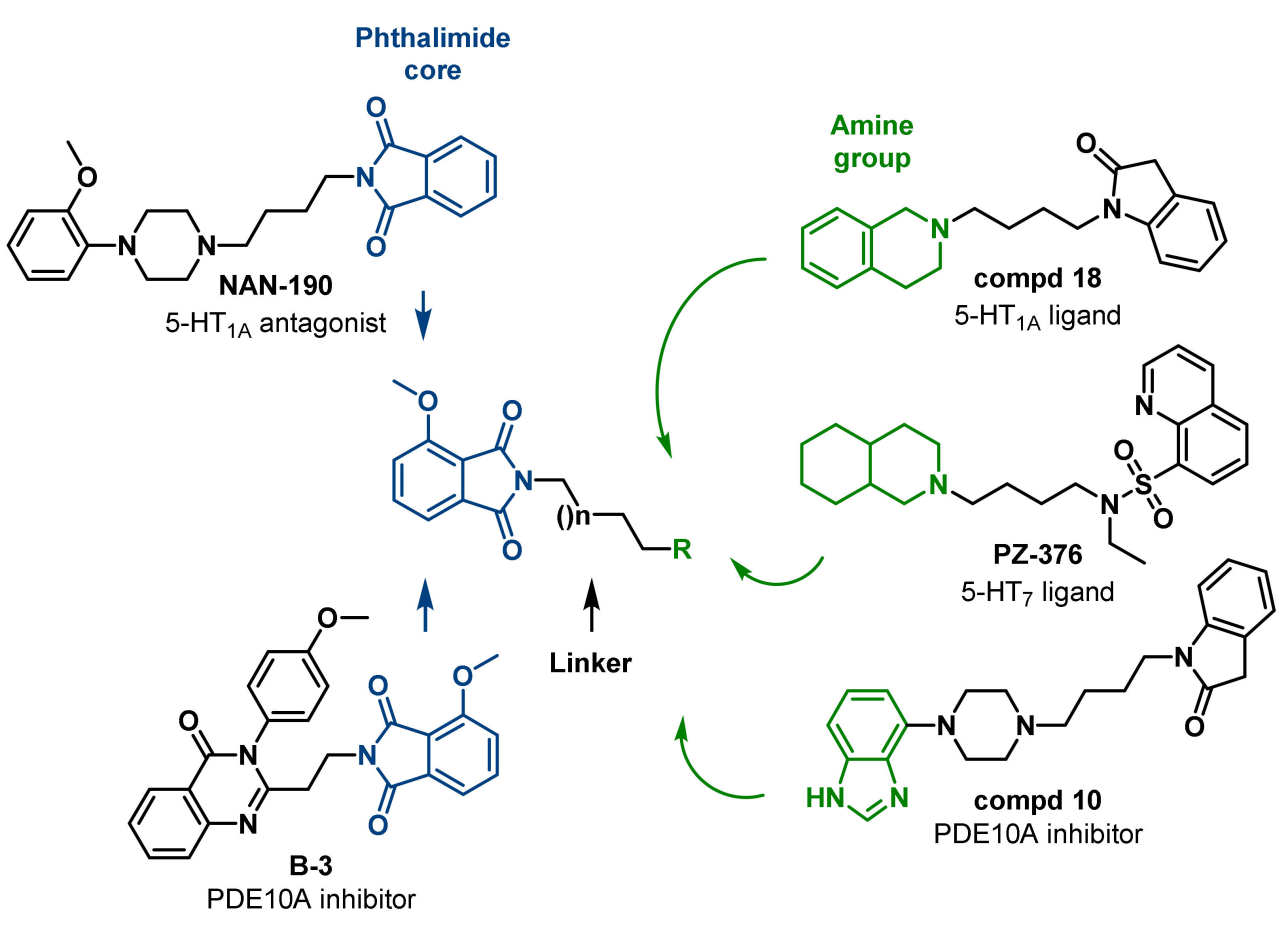
General structure of novel compounds **3**–**23**, designed based on potent phosphodiesterase 10A (PDE10) inhibitors and active serotonin receptor ligands [20,25,26,28,29,30]. The phthalimide cores and amine groups are highlighted in blue and green, respectively.

**Figure 3:**
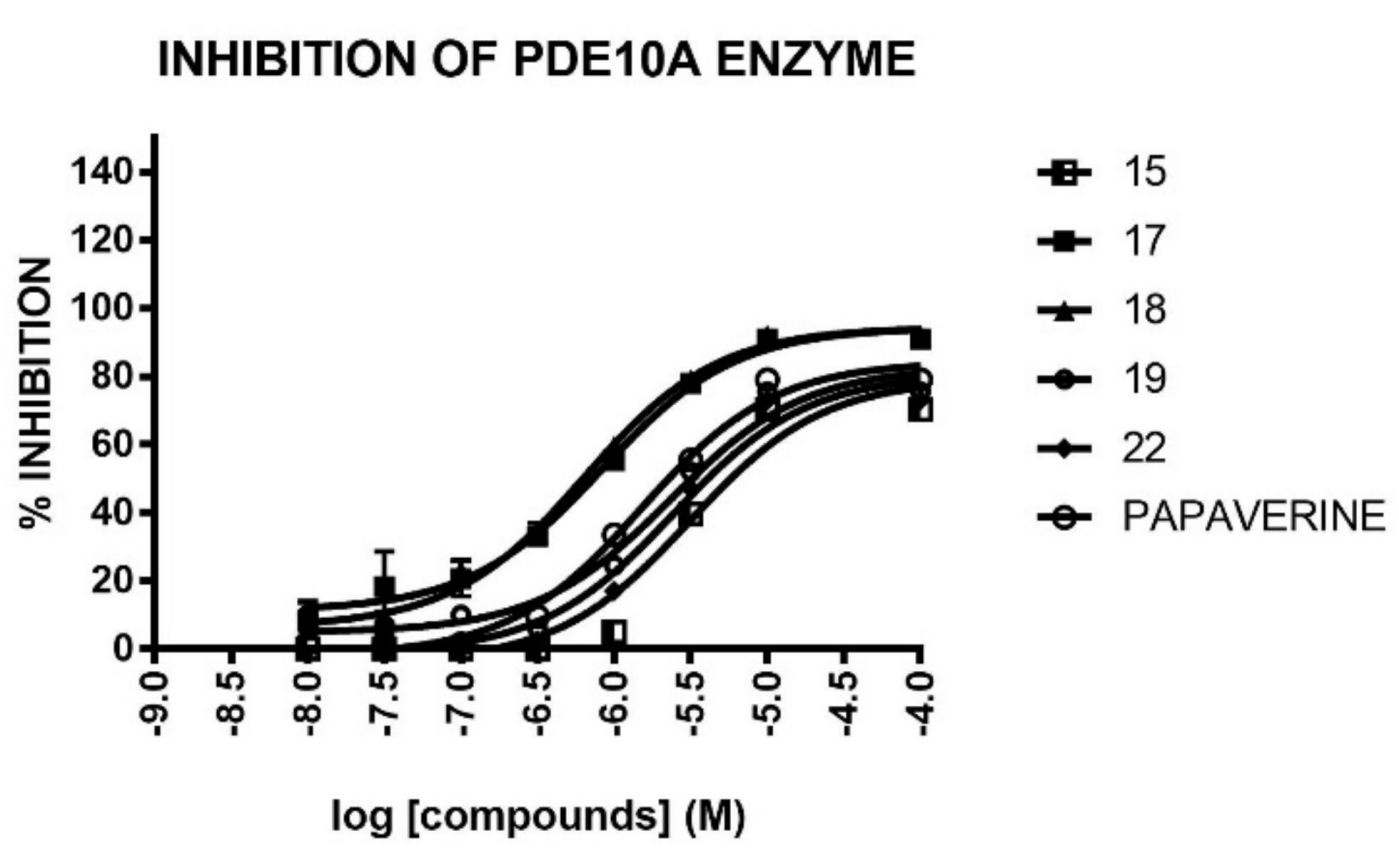
Dose-response curve of the most active compounds **15**, **17**, **18**, **19**, **22** and reference drug-papaverine on PDE 10A activity measured by the luminescence method.

**Figure 4:**
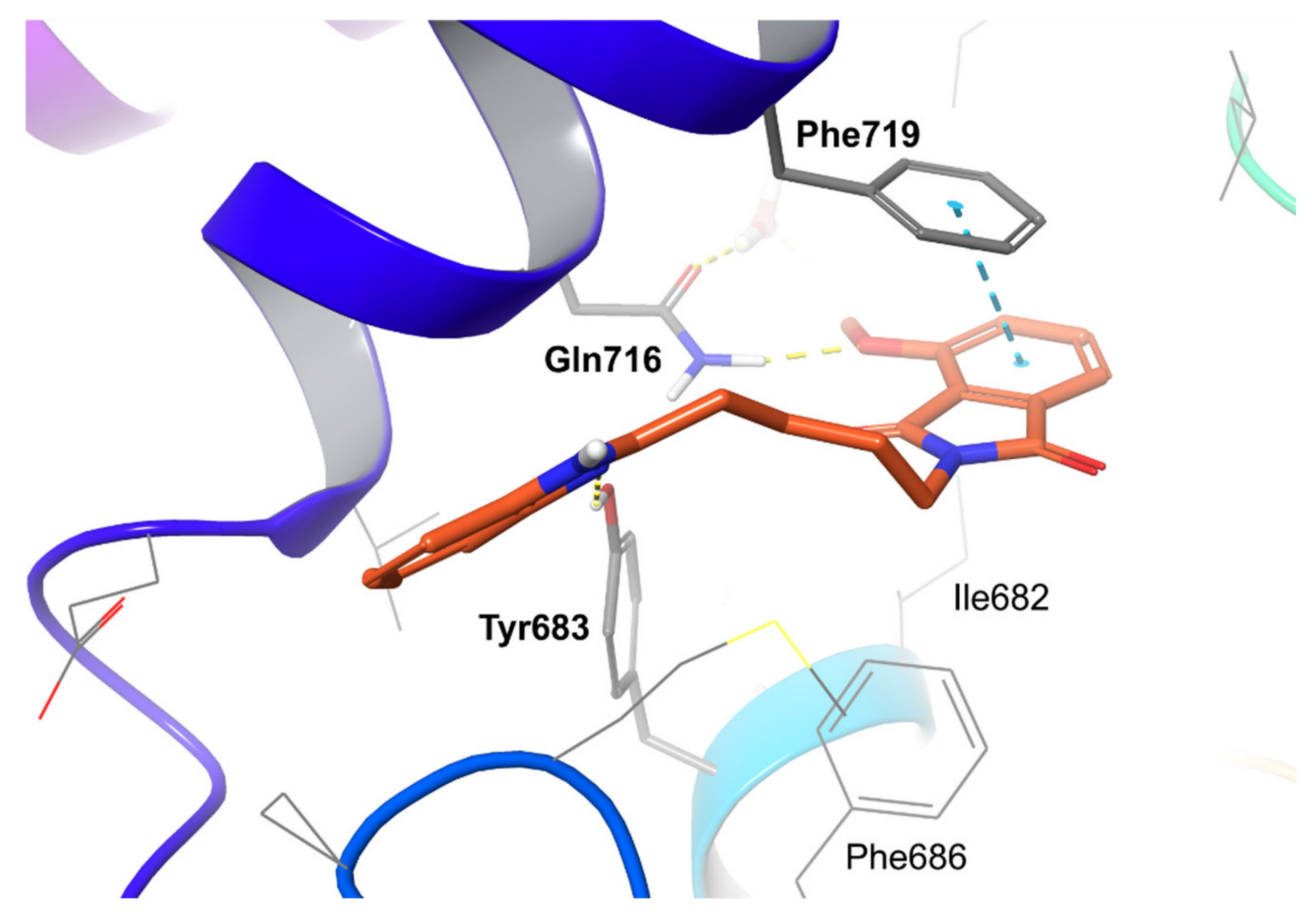
The predicted binding mode of compound **18** in the active site of phosphodiesterase 10A (PDB ID: 3SNI). Crucial interactions of the compound in the catalytic site with Gln716 and Phe719, as well as its positioning in the selectivity pocket (Tyr683) predict inhibitory activity of the series of rationally-designed derivatives synthesized in this study. Amino acid residues engaged in ligand binding (within 4 Å from the ligand atoms) are displayed as sticks, whereas crucial residues, e.g., forming H-bonds (dotted yellow lines) or π-π stacking (dotted cyan lines), are represented as thick sticks.

Isoindole-1,3-diones (phthalimides) and their N-substituted analogs have been a very interesting and topical research area due to their wide structural diversity and broad-spectrum biological activities. The sulphonamide and amide derivatives of *N*-phenyl-phthalimide (compounds **3a**–**e** and **4a**–**e**, Figure 1) displayed potent inhibitory activity on neutrophil recruitment, which correlated with their inhibitory effect on tumor necrosis factor α (TNF-α) level [1]. *N*-1-Adamantyl-4-aminophthalimide (compound **2′**, Figure 1) exhibits anti-HIV-1 and anti-HIV-2 activity in CEM cell cultures [2]. The N-pyridinyl and N-quinolinyl substituted derivatives of phthalimides (compounds **8′** and **12′**, Figure 1) demonstrated cytotoxicity against the growth of a number of cultured cell-lines [3]. 1,3-Dioxoisoindoline-5-carboxamides (compound **4d′**, Figure 1) are potent T-type calcium channel blockers [4], whereas 1,3-dioxoisoindoline-5-carboxylic acid derivatives (compounds **2c′** and **3c′**, Figure 1) are potent xanthine oxidase inhibitors [5]. Moreover, (*ZE*)-2-[4-(1-hydrazono-ethyl)phenyl]isoindoline-1,3-dione (compound **12′**, Figure 1) has shown remarkable anti-microbial activity [6]. In the central nervous system (CNS), N-phenylphthalimide derivatives (ADD213063 and ADD219048, Figure 1) have exhibited original anticonvulsant properties [7]. Pazinaclone (DN-2327, Figure 1) produces sedative and anxiolytic effects by acting as a partial agonist of GABA_A_ benzodiazepine receptors [8]. Potent acetylcholinesterase inhibitors (AChEIs) were identified in a group of 2-fluorinated-benzylamino-alkyl-isoindoline-1,3-diones [9], 2-(benzylamino-2-hydroxyalkyl)isoindoline-1,3-diones [10] (compounds **6′** and **12′**, Figure 1) and 2-(2-(4-benzoylpiperazin-1-yl)ethyl)isoindoline-1,3-dione (compound **4e′**, Figure 1) derivatives [11]. NAN-190 (2-methoxyphenyl)-4-(4-phthalimidobutyl)piperazine, Figure 2) is a selective antagonist of serotonin 1A subtype receptors (5-HT_1A_Rs) that potently blocks the alfa 2 adrenergic receptor (α_2_ARs) [12].

Schizophrenia is a multifactorial mental illness that affects 1% of the population. Both genetic and environmental contributions play an important role in the manifestation of a wide range of behavioral, emotional, and cognitive abnormalities in schizophrenia. Since the introduction of chlorpromazine, clinical practice has relied upon antipsychotic drugs (APDs) over the past half century, with numerous first-, second-, and third-generation antipsychotics discovered during this time. Typical, or first-generation, APDs (chlorpromazine and haloperidol) exhibited high-affinity dopamine D_2_ receptor antagonism, which causes both antipsychotic actions and many side effects e.g., extrapyramidal and endocrine. Atypical, or second generation, antipsychotics (SGAs) were then developed to avoid the extrapyramidal side effects. SGAs include compounds such as clozapine, olanzapine, and quetiapine that exhibit relatively moderate affinity for D_2_ and 5-HT_2A_ receptors or drugs like risperidone that effectively block both serotonin 5-HT_2A_ and dopamine D_2_ receptors [13,14]. The first representative of the third generation of APDs was aripiprazole, whose mechanism of action was ascribed to partial agonism of D_2_. Although aripiprazole has effects on several other receptors, this drug is the first “dopamine stabilizer” based on D_2_ partial agonist properties. However, the data available on the mechanism of action of aripiprazole are inconsistent with a mode of action related to the partial agonism of D_2_. Therefore, a recent hypothesis regarding the functional selectivity of aripiprazole for D_2_ has emerged that seems to describe its mechanism of action more accurately [15,16]. Although all approved antipsychotic drugs show affinity for dopamine D_2_ receptors, there is evidence that modulation of the serotonergic system leads to enhanced antipsychotic therapy [17,18]. Partial agonism of 5-HT_1A_, and antagonism of 5-HT_2A_, 5-HT_6_, and 5-HT_7_ receptors, contributes to a reduced risk of extrapyramidal side effects and improved specific cognition domains of atypical antipsychotic drugs [14].

The phosphodiesterase 10A (PDE10A) enzyme is highly expressed in the striatal medium spiny neurons (MSNs), where it modulates both dopamine D_2_- and D_1_-dependent signaling. Striatal MSNs pathways are divided into direct dopamine D_1_ receptor-mediated and indirect dopamine D_2_ receptor-mediated striatal output pathways [19]. Dopamine D_1_ receptors in direct pathway MSNs are coupled with G_s_ proteins, which activate adenylate cyclase to increase cyclic adenosine monophosphate (cAMP) concentration, whereas the stimulation of D_2_ receptors inhibits the activity of adenylyl cyclase, resulting in a decrease in the level of cAMP. On the other hand, inhibition of PDE10A in the dendritic membranes of MSNs results in suppression of D_2_-mediated signaling, which is similar to the effects of D_2_ antagonists. Upregulation of cyclic nucleotide levels in indirect pathway MSNs could be an alternative approach for novel antipsychotics [20,21], whereas activation of direct pathway MSNs could be associated with improvement of cognitive functions. Thus, in contrast to the marketed antipsychotics, phosphodiesterase inhibitors (PDEIs) influence both the indirect and direct pathways, and the additional action of these compounds on the direct pathway could help distinguish them clinically from D_2_ antagonists [22]. Moreover, PDEIs may have potential therapeutic utility in the treatment of cognitive impairment associated with schizophrenia or may be useful as an adjunctive treatment for negative symptoms in patients with schizophrenia [19].

In complex diseases such as schizophrenia, single-target drugs have turned out as failures, whereas multi-target drugs have been found to be much more efficacious. In clinical practice, many combinations of psychotropics have been tried for schizophrenia, based on a trial-and-error paradigm guided by clinical experience and patient response. An overall superior and safer solution seems to be the modern concept of designer multiple-targeting ligands (DMLs). The DMLs are also described as “polypharmacology by design”, which claims parallel modulation of defined multiple biological targets by designer small molecule drugs [23]. An exemplification of this idea is atypical antipsychotics, which target a number of G protein-coupled receptors (GPCRs) simultaneously. Currently, novel strategies in designing drugs effective in treatment of schizophrenia focus on targets beyond the dopaminergic hypothesis of the disease [24].

In order to search for novel potential antipsychotic agents, we rationally synthesized a series of N-substituted isoindole-1,3-dione derivatives, which were designed to interact with the serotonin 5-HT_1A_/5-HT_7_ receptor and/or inhibit PDE10A. The core 1*H*-isoindole-1,3(2*H*)-dione moiety was selected from the structure of the 5-HT_lA_ antagonist, NAN-190 (Figure 2). Next, a methoxy moiety was introduced at position 4 of the isoindole-1,3-dione so as to be able to obtain structural analogs of PDE10AIs (compound **B**-**3**, Figure 2) [25]. The isoindole-1,3-dione core was connected by one to five methylene linkers with amines. For determining the impact of N-alkylamine substitution of isoindole-1,3-dione on 5-HTR affinity and PDE10A inhibitory activity, different heterocyclic amine substituents were investigated. Modifications of amine substituents comprised changes in the size of the heterocyclic amine ring from 1*H*-imidazole and pyridine, to benzimidazole, 6,7-dimethoxy-1,2,3,4-tetrahydro-isoquinoline, 1,2,3,4-tetrahydroisoquinoline, and the decahydroisoquinoline moiety. Previously, these amines were used in the design of potent 5-HT_1A_ and 5-HT_7_ ligands (compound **18** and PZ-376, Figure 2) [26,27,28], and they are partially saturated analogs of isoquinoline, which is present in the structure of papaverine, a potent PDE10A inhibitor. Moreover, the benzimidazole moiety can be found in the structure of numerous PDE inhibitors and serotonin ligands (compound **10**, Figure 2) [29,30]. Finally, a phenyl ring was chosen instead of a heterocyclic amine group in order to examine the effect of amine substitution on selected 5-HTRs affinity and PDE10A inhibitory activity.

The series of designed ligands were synthesized and evaluated in adequate biological assays, a luminescence PDE inhibition assay, as well as a radioligand binding assay for 5-HT_1A_ and 5-HT_7_ receptors. Computer-aided studies were undertaken to identify the structural elements responsible for in vitro activity. Finally, for the most active compound, Compound **18**, in vitro safety studies were performed and preliminary in vivo activity was tested in an animal model of schizophrenia.

## 2. Results and Discussion

### 2.1. Chemistry

**Scheme 1:**
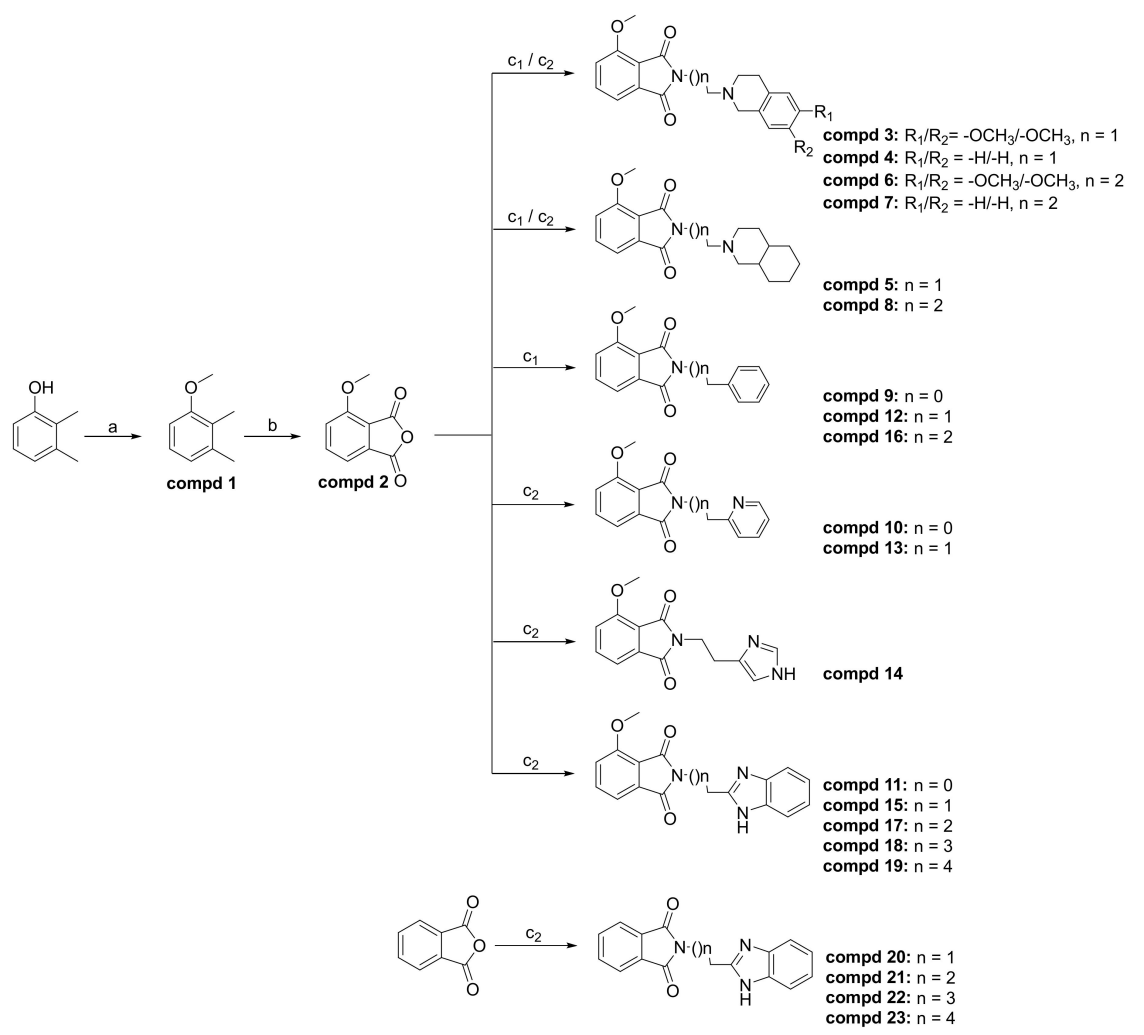
Reagents and conditions of the chemical synthesis route: (a) CH_3_I, K_2_CO_3_, DMF, 75 °C, 12 h; (b) KMnO_4_, H_2_O/tBuOH, reflux, 12 h; AcOH, THF, reflux, 4 h; (c_1_) appropriate amine, AcOH, reflux, 48 h; (c_2_) appropriate amine, TEA, 4Å molecular sieves, toluene, reflux, 16 h. 2,3-Dimethylphenol and phthalic anhydride were commercially available.

**Scheme 2:**
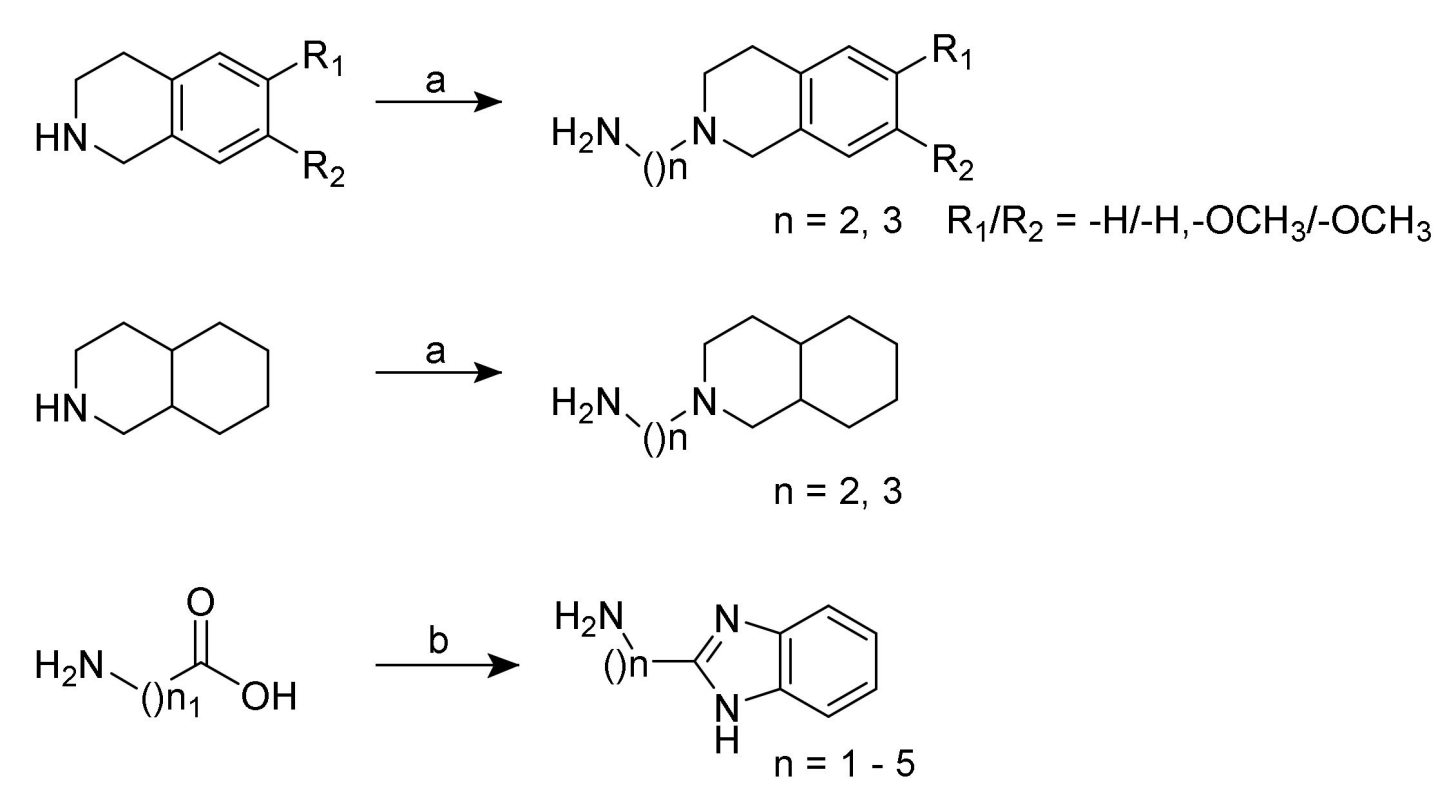
Reagents and conditions of the chemical substitution routes: (**a**) appropriate amine, *N*-bromoalkylphthalimide, K_2_CO_3_, MeCN, reflux, 12 h; 40% CH_3_NH_2_, 20% NaOH, room temperature, 2 h; (**b**) appropriate amino acid, 1,2-phenylendiamine, 4N HCl, microwave heating, 100 °C, 40 min [33,34].

The series of rationally designed 4-methoxy-1*H*-isoindole-1,3(*2H*)-dione derivatives (**3**–**23**) were synthesized in average to good yields (30–75%), as shown in Scheme 1. The starting 2,3-dimethylphenyl methyl ether (**1**) was synthesized by a procedure published elsewhere [31]. The intermediate compound, 4-methoxy-2-benzofuran-1,3-dione (**2**), was obtained from **1**, according to a previously described procedure [32] with slight modifications. The aminoalkylamine moieties derived from 6,7-dimethoxy-1,2,3,4-tetrahydroisoquinoline, 1,2,3,4-tetrahydroisoquinoline, decahydroisoquinoline, and 1*H*-1,3-benzodiazole were obtained in line with the earlier reported methods (Scheme 2) [33,34], while those containing 1*H*-imidazole, pyridine, and phenyl rings were commercially available.

### 2.2. In Vitro Activity of Compounds ***3***–***23***

#### 2.2.1. PDE10A Inhibitory Activity

The novel series of compounds was derived from a 4-methoxy-2,3-dihydro-1*H*-isoindole-1,3-dione (4-methoxyphthalimide, Figure 1) fragment, which was connected via different lengths of a carbon-chain linker (C_1_–C_5_) to various amine fragments (e.g., 6,7-dimethoxy-1,2,3,4-tetrahydroisoquinoline; 1*H*-imidazole; 1,2,3,4-tetrahydroisoquinoline; 1*H*-benzimidazole; and decahydroisoquinoline) or to a phenyl ring (**3**–**17**). In this series, seven compounds (**7**, **8**, **11**, **12**, **15**–**17**) displayed PDE10A inhibitory activity in the luminescence PDE inhibition assay at a concentration of 10 µM (7–92%, Table 1). Furthermore, four analogs of the most promising 4-methoxyphthalimide derivatives (**15**, **17**–**19**), containing the phthalimide moiety, were synthesized (**20**–**23**). Among them, three of the four phthalimide derivatives (**21**–**23**) showed moderate activity toward PDE10A (45–73%, Table 1).

Initially, among compounds with a linker length of one to three methylene units (**3**–**17**), the highest percentage of PDE10A inhibition (70–86%) was exhibited by compounds containing the 1*H*-benzimidazole moiety (**15**, **17**). Therefore, the influence of the length of the linker on inhibition of PDE10A was investigated in this group of compounds. In the case of the 1*H*-benzimidazole containing compounds, **11**, **15**, and **17**–**19**, the elongation of the linker from one to five methylene groups resulted in an increase and then a decrease in the inhibition of PDE10A. The most active compound among them, compound **18** (92% inhibition), has a linker of four methylene units that connects the 4-methoxyphthalimide fragment with the 1*H*-benzimidazole moiety. The modifications of the 1*H*-benzimidazole moiety, such as replacement with 1,2,3,4-tetrahydroisoquinoline fragment (**4**, **7**), or its saturation (**8** vs. **7**), as well as removal of one aromatic ring in this part (**10**, **13, 14**) decreased the observed affinity to PDE10A. Interestingly, compounds **12** and **16,** containing a phenyl moiety at the terminal location, despite the lack of a functional amine group, showed very low activity toward PDE10A.

In addition, the relevance of the introduction of a 4-methoxyl group to the 4-methoxy-2,3-dihydro-1*H*-isoindole-1,3-dione fragment was also verified. For this purpose, a small phthalimide series without a 4-methoxy group at position 4 (Table 1) connected via an alkylene linker to the 1*H*-benzimidazole moiety (**20**–**23**) was synthesized. The activity of compounds **20**–**23** was significantly lower than that of their 4-methoxy analogs (**15**, **17**–**19**). Moreover, similar to the 4-methoxy series, the inhibitory activity of compounds **20**–**23** increased with the length of the linker up to four methylene units and then slightly decreased.

In the next step, the five most active compounds chosen (**15**, **17**–**19**, **22**) were quantitatively tested in the luminescence PDE inhibition assay, alongside papaverine as the reference drug. The effectiveness of inhibition was expressed as the half maximal inhibitory concentration (IC_50_) values. The chosen compounds displayed a percentage of inhibition above 70% at the concentration of 10 µM. All investigated compounds (**15**, **17**–**19**, **22**) displayed satisfactory IC_50_ values (886 nM–6.71 µM, Table 1 and Figure 3) in comparison with papaverine (5.755 µM). It is noteworthy that the most potent PDE10A inhibitor, compound **18** (IC_50_ = 886 nM), was in-line with the predictions stipulated by Lipinski’s Rule of Five, which describes “drug-like” parameters of small molecules in terms of lipophilicity, size, polarity, solubility, flexibility, and saturation [35] (see graph, Figure S1, in Supporting Materials).

#### 2.2.2. Serotonin Receptor Affinity

In the next phase of the current study, the binding affinities of novel 4-methoxy-2,3-dihydro-1*H*-isoindole-1,3-dione derivatives for 5-HT_1A_ and 5-HT_7_ receptors were determined in a radioligand binding assay (Table 1). The results revealed that the majority of the tested compounds displayed very low-to-moderate affinity for the 5-HT_1A_ receptor (6–59%) at a concentration of 1 µM, but only a few showed any affinity for the 5-HT_7_ receptor (**7**, **16**–**23**). The highest 5-HT_1A_ or 5-HT_7_ receptor affinity was observed for compounds **13** (59% for 5-HT_1A_) and **7** (51% for 5-HT_7_), respectively. Among the investigated series, three compounds (**7**, **20**, and **23**) showed a low but significant (above 20%) percentage of binding for both 5-HT_1A_ and 5-HT_7_ receptors (33%, 26%, 48% and 51%, 36%, 34%, respectively).

In summary, the present study determined that the majority of the 2,3-dihydro-1*H*-isoindole-1,3-dione derivatives (**3**–**23**) showed affinity toward the 5-HT_1A_ receptor, whereas only few bind at the 5-HT_7_ receptor site. In the case of 1*H*-benzimidazole derivatives (compounds **15**, **17**–**19**), which significantly inhibited PDE10A (IC_50_ = 886 nM–6.71 µM), low affinity for 5-HT_1A_ receptors (16–44%), and even lower for 5-HT_7_ receptors (0–10%) was observed.

Furthermore, because compound **18** contains structural moieties such as benzimidazole and phthalimide, which are also present in serotonin 5-HT_2A_ and dopamine D_2_ receptor antagonists **[14]**, it was decided to test the affinity of Compound **18** to these receptors. The obtained results (appendix below Table 1) revealed that at a concentration of 1 µM compound **18** displayed negligible percentage of binding for 5-HT_2A_ and none for D_2_ receptors (8.8% and 0%, respectively).

### 2.3. Molecular Modeling Studies

In line with in vitro results, the most active 4-methoxy-2,3-dihydro-1*H*-isoindole-1,3-dione derivative with a bezimidazol-2-yl butyl moiety (**18**) was selected to clarify and draw useful information regarding the nature of the interactions of PDE10A with its ligands, and insights into structure-activity relationships were investigated. We elucidated putative binding modes of several derivatives throughout the process of docking to the protein target, which was represented by the optimized crystal structure of the studied enzyme. As a representative example, binding interactions were presented for compound **18** (2-[4-(1*H*-1,3-benzodiazol-2-yl)butyl]-4-methoxy-2,3-dihydro-1*H*-isoindole-1,3-dione) (Figure 4).

The 2,3-dihydro-1*H*-isoindole-1,3-dione moiety, bound in the catalytic active site, interacted with Gln716 (H-bond) and with Phe719 (π-π stacking). In the case of the most active compound (**18**), the 4-methoxy group acted as the H-bond acceptor, whereas unsubstituted analogs interacted through the carbonyl group of the imide moiety. The oxygen atom of the phthalimide was less electronegative than the one of the methoxy group, thus making the H-bond accepting properties weaker and disadvantageous in comparison with that of the 4-methoxy-substituted analogs. Moreover, the H-bond formed by the latter group attracted the heteroaryl ring of the ligand closer to Phe719/686, thereby facilitating the π-π interaction in the hydrophobic clamp. The 1*H*-1,3-benzodiazole fragment accepted the H-bond from Tyr683 in the selectivity pocket. The interaction was secured by the optimal length of the linker—4 carbon atoms and resulted in favorable scoring function value −8.885 (glide gscore) (Figure 4). Both longer and shorter aliphatic chains decreased the inhibitory potential (see Table 1), enforcing suboptimal arrangement of the terminal moieties in the active site, which resulted in the loss of one of the aforementioned key interactions (e.g., in the case of compound **19**) or decrease in scoring function value (−8.254 for compound **15**).

### 2.4. Cytotoxicity

In the subsequent stage of the study, the safety profile of the most active compound, **18**, was analyzed. Such analysis requires the exclusion of adverse effects of organ toxicity before proceeding to further in vivo studies. According to the guidelines for the study of biologically active molecules, cell lines are a good alternative to animal models at the initial stage of the drug cytotoxicity evaluation process. The two cell lines used in this study, namely, the SHSY-5Y and HepG2 cell lines are approved in vitro models, which can be used to obtain preliminary results regarding possible neuro- and hepato-toxicity [36].

**Figure 5:**
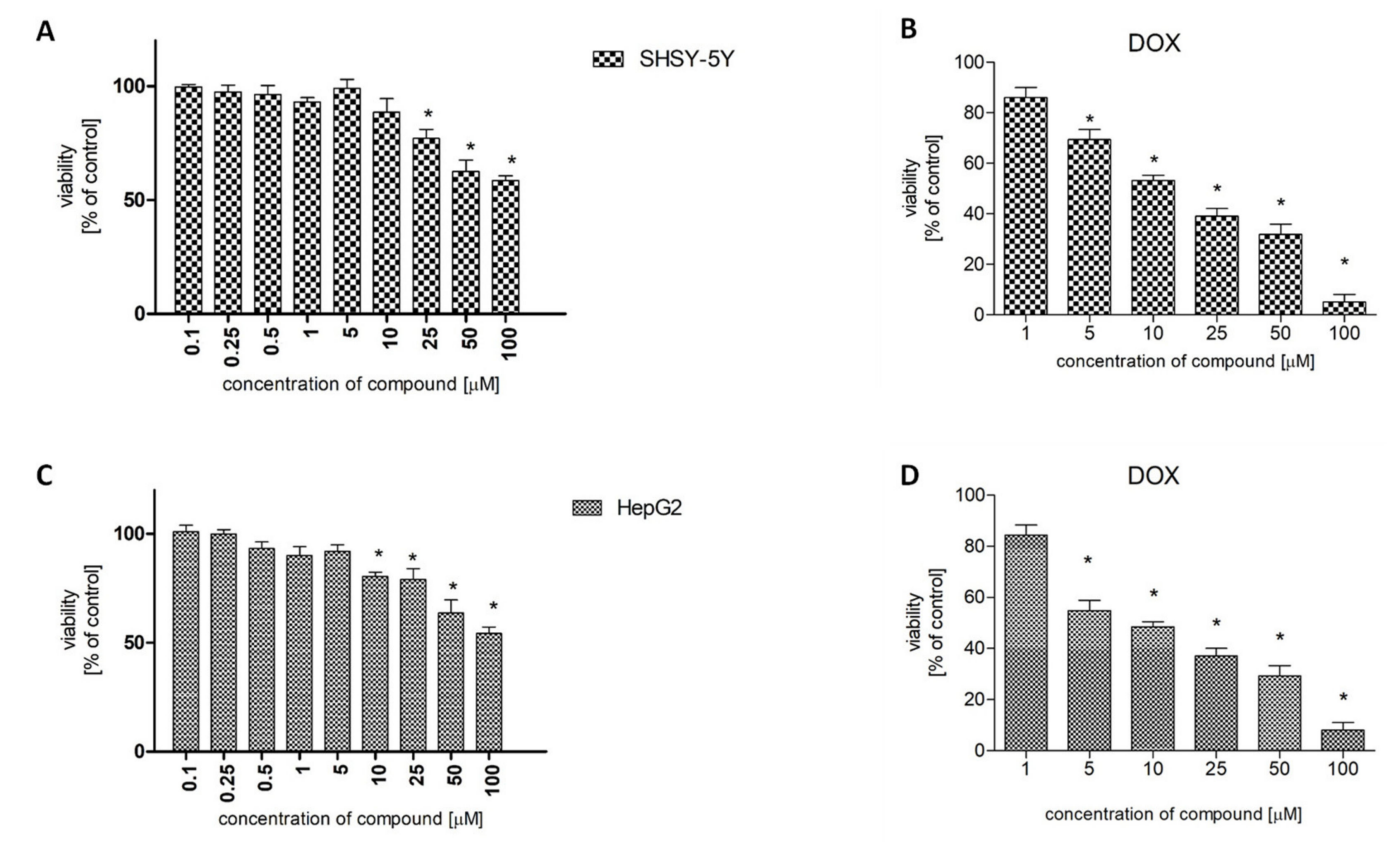
Viability of SHSY-5Y and HepG2 cells treated with compound **18** in the MTT test; doxorubicin (DOX) was used as reference control. (**A**): compound **18** (SHSY-5Y), (**B**): DOX (SHSY-5Y), (**C**): compound **18** (HepG2), (**D**): DOX (HepG2). Graphs represent the viability of cells as a % of control (non-treated cells). Values represent means ± SEM of three independent experiments. Statistical significance (*) vs. control was determined using the Mann-Whitney test, *p* < 0.05.

The MTT assay, which measures the metabolic status of cells, was used to determine cytotoxicity. The results of the experiments clearly indicate that compound **18** is safe. Decreasing cell viability (HepG2/SHSY-5Y) is observed only at higher concentrations of compound **18**, while the reference standard, doxorubicin (DOX), a well-described cytotoxic agent, demonstrated significantly decreased cell viability already at much lower concentrations (Figure 5).

### 2.5. Behavioral Evaluation

**Figure 6:**
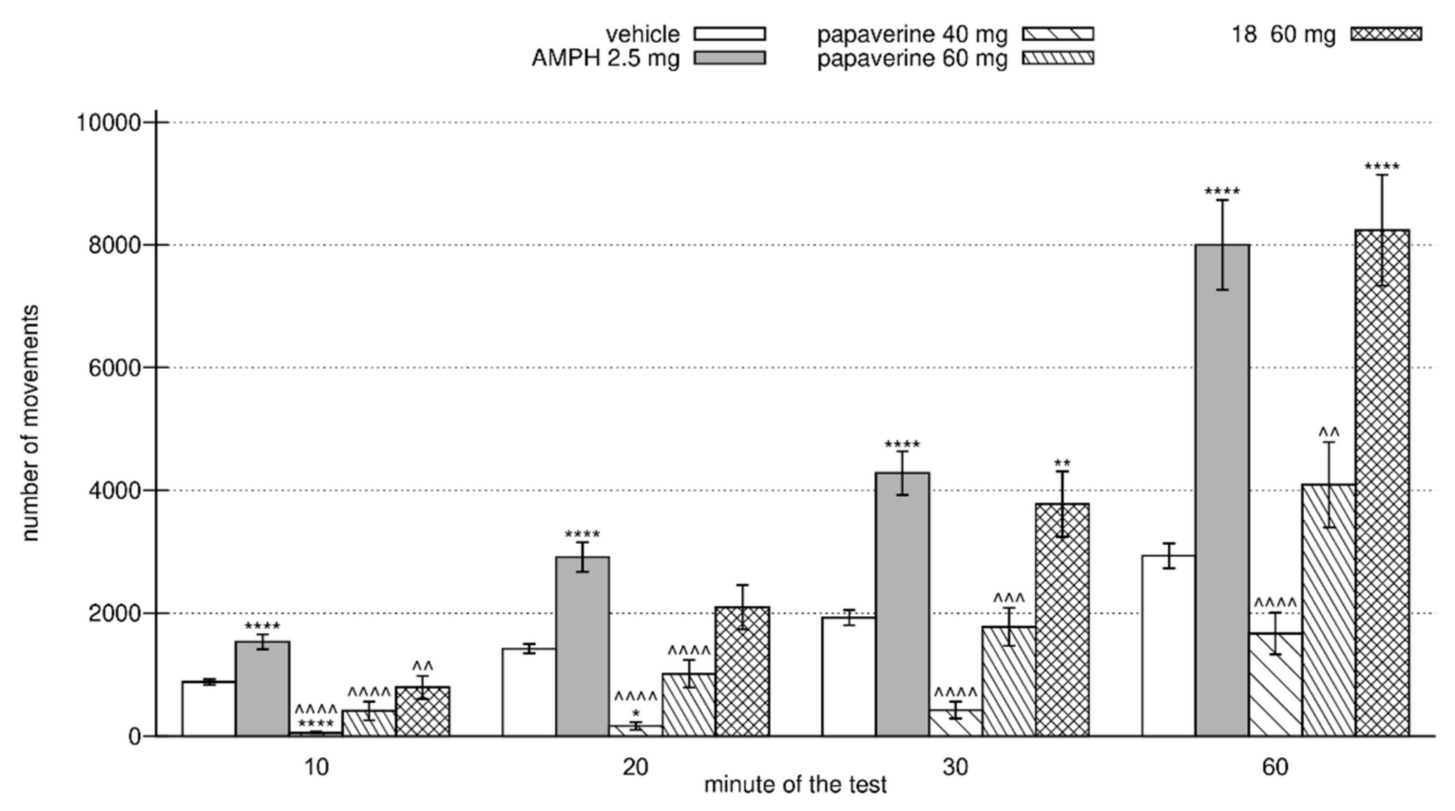
Effects of papaverine and compound **18** on *d*-amphetamine-induced hyperlocomotor activity in CD-1 mice. Data are expressed as the mean of the number of movements recorded after 10, 20, 30, and 60 min of the papaverine-treated groups (at doses of 40 and 60 mg, respectively) and the compound **18**-treated group (at a dose of 60 mg) ± S.E.M. * *p* < 0.05, ** *p* < 0.01, **** *p* < 0.0001, versus the respective vehicle-treated group, ^^ *p* < 0.01, ^^^ *p* < 0.001, ^^^^ *p* < 0.0001, and versus the respective AMPH-treated group (one way ANOVA followed by Bonferroni’s post-hoc test). AMPH-*d*-amphetamine.

For further behavioral characterization, compound **18**, with the highest PDE10A inhibitory activity and satisfactory cytotoxicity profile, was selected. Thus, the potential antipsychotic properties of compound **18** as an inhibitory effect on PDE10A in a behavioral animal model was determined. The obtained results revealed that treatment of mice with d-amphetamine (AMPH) significantly increased horizontal locomotor activity throughout the duration of the test. Papaverine dose-dependently antagonized this response at both tested doses of 40 and 60 mg/kg for 60 min. Compound **18** administered at a dose of 60 mg/kg decreased AMPH-induced hyperactivity, but only for the first 10 min after injection (Figure 6). The lower doses of compound **18**, as well as papaverine, did not change the stimulating effect of AMPH (data not shown). To assess if a nonspecific suppression of locomotion contributes to the ability of the tested compounds to oppose AMPH-induced hyperactivity, the influence of these compounds on spontaneous locomotor activity was measured. The locomotor-suppressing effects were observed after the administration of papaverine (40 and 60 mg/kg) as well as **18** (60 mg/kg) (see Table S1 in Supporting Materials).

In summary, the suppression of AMPH-induced hyperlocomotion suggests antipsychotic-like properties of compound **18,** as well as of papaverine. However, these effects are transient and may only reflect the decrease in spontaneous locomotor activity. Our findings are consistent with previous studies conducted with papaverine in rats [19,37], showing that the period of papaverine-induced suppression of spontaneous locomotor activity fully included time of reversal of hyperlocomotion produced by AMPH injection. Similar findings are reported by Schmidt et al. [38] for compound TP-10, which is a PDE-10 inhibitor with improved potency and selectivity. The aforementioned results suggest that antipsychotic-like properties of PDE10A inhibitors detected with locomotor-based paradigms need to be treated with caution, due to concurrent inhibition of spontaneous locomotor activity. Moreover, at this stage of our preliminary study, we can only speculate that the cause of compound **18**′s transient effect on AMPH-induced hyperlocomotor activity may be a result of either rapid redistribution to the other brain structures or rapid metabolism. In order to overcome possible pharmacokinetic and metabolic stability problems, we plan to further optimize the leading structure of compound **18** and conduct extended in vitro and in vivo investigations.

## 3. Materials and Methods

### 3.1. Chemistry

All chemicals and solvents were purchased from commercial suppliers (Aldrich, Poznań, Poland and Chempur, Piekary Śląskie, Poland) and were used without further purification. Melting points were measured in open capillaries on an Electrothermal 9300 apparatus. Thin-layer chromatography (TLC) was run on Merck silica gel 60 F_254_ aluminium sheets (Merck; Darmstadt, Germany), using the following mixtures of solvents: (S_1_) dichloromethane (9)/methanol (0.3), (S_2_) dichloromethane (9)/methanol (0.7), (S_3_) dichloromethane (9)/methanol (1). Analytical HPLC was conducted on a Waters HPLC instrument with a Waters 485 Tunable Absorbance Detector UV, equipped with a Symetry column (C18, 3.5 µm, 4.6 × 30 mm) using a water/acetonitrile gradient with 0.1% trifluoroacetic acid (TFA) as the mobile phase at a flow rate of 5 mL/min.

Additionally, the liquid chromatography/mass spectrometry (LC/MS) analysis was performed on a Waters Acquity TQD system, with a Waters TQD quadrupole mass spectrometer with detection by UV (DAD) using an Acquity UPLC BEH C18 column (1.7 μm, 2.1 mm × 100 mm). A water/acetonitrile gradient with 0.1% TFA was used as a mobile phase at a flow rate of 0.3 mL/min. The UPLC/MS purity of the investigated compounds (**3**–**23**) proved to be over 98%. NMR spectra were recorded on a Varian Mercury 300 MHz spectrometer (Varian Inc., Palo Alto, CA, USA) using the solvent (CDCl_3_ or DMSO-*d*_6_) signal as an internal standard; chemical shifts are expressed in parts per million (ppm). Signal multiplets are represented by the following abbreviations: s (singlet), brs (broad singlet), d (doublet), t (triplet), m (multiplet).

#### 3.1.1. General Procedure for Synthesis of 4-Methoxy-2-benzofuran-1,3-dione (2)

The 4-methoxy-2-benzofuran-1,3-dione was obtained from **1**, according to a previously published procedure [32], with slight modifications. Briefly, 2,3-dimethylphenyl methyl ether (3.4 g, 25 mmol) was dissolved in water (98 mL) and tert-butanol (42 mL). After potassium permanganate (25 g, 160 mmol) was added, the reaction mixture was refluxed for 12 h. The resulting suspension was filtered over Celite, concentrated to 50 mL, acidified with 37% hydrochloric acid, and left standing overnight at room temperature. The 3-methoxybenzene-1,2-dicarboxylic acid crystallized as a white solid (4.34 g) and was refluxed for 4 h in anhydrous tetrahydrofuran (24 mL) with the addition of acetic anhydride (7 mL). Upon cooling to room temperature, the reaction mixture was concentrated in vacuo and the white solid was dried at 50 °C for 48 h, to afford 4-methoxy-2-benzofuran-1,3-dione (3.82, 97%). HPLC: *R_t_* = 0.931, MS calcd for [M + H]^+^: C_9_H_6_O_4_ *m/z*: 178.14, found: 178.97; ^1^H-NMR (300 MHz, DMSO-*d*_6_) δ ppm 3.99 (s, 3 H) 7.59 (dd, *J* = 9.67, 7.91 Hz, 2 H) 7.90–7.97 (m, 1 H).

#### 3.1.2. General Procedure for Synthesis of the Final Series of Compounds (**3**–**23**)

The final compounds (**3**–**23**) were obtained within two different pathways, described in procedures A and B. Procedure A (**3**–**5**, **9**, **12**, **16**): a stoichiometric amount of 4-methoxy-2-benzofuran-1,3-dione (0.196 g, 1.1 mmol) and appropriate amine (1.1 mmol) in glacial acetic acid (2 mL) was refluxed for 48 h, with stirring. Upon cooling to room temperature, the reaction mixture was extracted with diethyl ether and concentrated in vacuum [39]. Procedure B (**6**–**8**, **10**, **11**, **13**–**15**, **17**–**23**): an amount of 4-methoxy-2-benzofuran-1,3-dione (0.178 g, 1.0 mmol), along with specific amounts of an appropriate amine (1.2 mmol), trimethylamine (0.28 mL, 2 mmol), and 4 Å molecular sieves were added in anhydrous toluene (4 mL) and the mixture was refluxed and stirred overnight. The reaction mixture was then filtered and the solvent was evaporated in vacuum [40]. All the final compounds (**3**–**23**) were purified by column chromatography using dichloromethane (9)/methanol (0.7) as eluent.

##### 2-[2-(6,7-Dimethoxy-3,4-dihydroisoquinolin-2(1*H*)-yl)ethyl]-4-methoxy-1*H*-isoindole-1,3(2*H*)-dione (**3**)

Yellow powdery crystals. Yield: 42%; mp 108–109 °C; TLC: *R_f_* = 0.44 (S_1_); HPLC: *t_R_* = 0.979; MS calcd for [M + H]^+^: C_22_H_24_N_2_O_5_ *m/z*: 396.17, found: 397.16; ^1^H-NMR (300 MHz, CDCl3-*d*) δ ppm 2.72–2.82 (m, 6 H) 3.62 (s, 2 H) 3.78–3.82 (s, 6 H) 3.84–3.90 (m, 2 H) 3.99 (s, 3 H) 6.52 (d, *J* = 16.16 Hz, 2 H) 7.12–7.20 (m, 1 H) 7.34–7.43 (m, 1 H) 7.61 (dd, *J* = 8.46, 7.44 Hz, 1 H).

##### 2-[2-(3,4-Dihydroisoquinolin-2(1*H*)-yl)ethyl]-4-methoxy-1*H*-isoindole-1,3(2*H*)-dione (**4**)

White powdery crystals. Yield: 75%; mp 157–158 °C; TLC: *R_f_* = 0.27 (S_1_); HPLC: *t_R_* = 0.906; MS calcd for [M + H]^+^: C_20_H_20_N_2_O_3_ *m/z*: 336.38, found: 337.14; ^1^H-NMR (300 MHz, CDCl_3_-*d*) δ ppm 2.76–2.87 (m, 6 H) 3.70 (s, 2 H) 3.88 (t, *J* = 6.54 Hz, 2 H) 3.99 (s, 3 H) 6.96–7.02 (m, 1 H) 7.04–7.11 (m, 3 H) 7.15 (d, *J* = 8.46 Hz, 1 H) 7.39 (d, *J* = 7.44 Hz, 1 H) 7.61 (dd, *J* = 8.46, 7.18 Hz, 1 H).

##### 2-[2-(Octahydroisoquinolin-2(1*H*)-yl)ethyl]-4-methoxy-1*H*-isoindole-1,3(2*H*)-dione (**5**)

Creamy powdery crystals. Yield: 35%; mp 145–146 °C; TLC: *R_f_* = 0.55 (S_2_); HPLC: *t_R_* = 1.124; MS calcd for [M + H]^+^: C_20_H_26_N_2_O_3_ *m/z*: 342.43, found: 343.19; ^1^H-NMR (300 MHz, CDCl_3_-*d*) δ ppm 0.79–1.00 (m, 3 H) 1.08–1.30 (m, 4 H) 1.45–1.71 (m, 6 H) 1.94–2.05 (m, 1 H) 2.57 (t, *J* = 7.05 Hz, 2 H) 2.79–2.86 (m, 1 H) 2.99 (d, *J* = 10.77 Hz, 1 H) 3.78 (t, *J* = 7.05 Hz, 2 H) 4.01 (s, 3 H) 7.18 (d, *J* = 8.21 Hz, 1 H) 7.42 (dd, *J* = 7.31, 0.64 Hz, 1 H) 7.64 (dd, *J* = 8.34, 7.31 Hz, 1 H).

##### 2-[3-(6,7-Dimethoxy-3,4-dihydroisoquinolin-2(1*H*)-yl)propyl]-4-methoxy-1*H*-isoindole-1,3(2*H*)-dione (**6**)

Creamy powdery crystals. Yield: 39%; mp 107–109 °C; TLC: *R_f_* = 0.38 (S_2_); HPLC: *t_R_* = 1.018; MS calcd for [M + H]^+^: C_23_H_26_N_2_O_5_ *m/z*: 410.18, found: 411.18; ^1^H-NMR (300 MHz, CDCl_3_-*d*) δ ppm 1.92 (quin, *J* = 6.86 Hz, 2 H) 2.53–2.71 (m, 6 H) 3.45 (s, 2 H) 3.72–3.84 (m, 8 H) 3.96 (s, 3 H) 6.45 (d, *J* = 5.90 Hz, 2 H) 7.08 (d, *J* = 8.46 Hz, 1 H) 7.35 (d, *J* = 7.18 Hz, 1 H) 7.56 (dd, *J* = 8.34, 7.31 Hz, 1 H).

##### 2-[3-(3,4-Dihydroisoquinolin-2(1*H*)-yl)propyl]-4-methoxy-1*H*-isoindole-1,3(2*H*)-dione (**7**)

Yellow powdery crystals. Yield: 48%; mp 92–93 °C; TLC: *R_f_* = 0.32 (S_2_); HPLC: *t_R_* = 1.006; MS calcd for [M + H]^+^: C_21_H_22_N_2_O_3_ *m/z*: 350.41, found: 351.36; ^1^H-NMR (300 MHz, CDCl_3_-*d*) δ ppm 1.94 (quin, *J* = 6.92 Hz, 2 H) 2.58 (t, *J* = 7.05 Hz, 2 H) 2.63–2.69 (m, 2 H) 2.74–2.81 (m, 2 H) 3.55 (s, 2 H) 3.77 (t, *J* = 6.92 Hz, 2 H) 3.96 (s, 7 H) 3.93–3.99 (m, 3 H) 6.92–7.12 (m, 5 H) 7.36 (dd, *J* = 7.18, 0.51 Hz, 1 H) 7.56 (dd, *J* = 8.46, 7.18 Hz, 1 H).

##### 2-[3-(Octahydroisoquinolin-2(1*H*)-yl)propyl]-4-methoxy-1*H*-isoindole-1,3(2*H*)-dione (**8**)

Creamy powdery crystals. Yield: 46%; mp 103–104 °C; TLC: *R_f_* = 0.47 (S_3_); HPLC: *t_R_* = 1.161; MS calcd for [M + H]^+^: C_21_H_28_N_2_O_3_ *m/z*: 356.21, found: 357.21; ^1^H-NMR (300 MHz, CDCl_3_-*d*) δ ppm 0.78–0.93 (m, 3 H) 1.12–1.28 (m, 4 H) 1.43–1.71 (m, 6 H) 1.86–1.99 (m, 3 H) 2.48 (t, *J* = 7.44 Hz, 2 H) 2.82 (d, *J* = 9.23 Hz, 1 H) 3.00 (d, *J* = 11.03 Hz, 1 H) 3.69 (t, *J* = 6.80 Hz, 2 H) 4.00 (s, 3 H) 7.17 (d, *J* = 8.21 Hz, 1 H) 7.40 (dd, *J* = 7.31, 0.64 Hz, 1 H) 7.63 (dd, *J* = 8.46, 7.18 Hz, 1 H).

##### 2-Benzyl-4-methoxy-1*H*-isoindole-1,3(2*H*)-dione (**9**)

White powdery crystals. Yield: 75%; mp 167–168 °C; TLC: *R_f_* = 0.70 (S_1_); HPLC: *t*_R_ = 1.435; MS calcd for [M + H]^+^: C_16_H_13_NO_3_ *m/z*: 267.09, found: 268.09; ^1^H-NMR (300 MHz, CDCl_3_-*d*) δ ppm 4.00 (s, 3 H) 4.81 (s, 2 H) 7.17 (d, *J* = 7.95 Hz, 1 H) 7.21–7.33 (m, 3 H) 7.40–7.46 (m, 3 H) 7.64 (dd, *J* = 8.46, 7.44 Hz, 1 H).

##### 4-Methoxy-2-(pyridin-2-ylmethyl)-1*H*-isoindole-1,3(2*H*)-dione (**10**)

Creamy powdery crystals. Yield: 53%; mp 144–145 °C; TLC: *R_f_* = 0.56 (S_2_); HPLC: *t_R_* = 0.755; MS calcd for [M + H]^+^: C_15_H_12_N_2_O_3_ *m/z*: 268.08, found: 269.09; ^1^H-NMR (300 MHz, CDCl_3_-*d*) δ ppm 4.00 (s, 3 H) 4.97 (s, 2 H) 7.10–7.17 (m, 1 H) 7.18–7.28 (m, 2 H) 7.46 (d, *J* = 7.95 Hz, 1 H) 7.56–7.69 (m, 2 H) 8.50 (dd, *J* = 4.87, 2.56 Hz, 1 H).

##### 2-(1*H*-Benzimidazol-2-ylmethyl)-4-methoxy-1*H*-isoindole-1,3(2*H*)-dione (**11**)

Creamy powdery crystals. Yield: 30%; mp 239–240 °C; TLC: *R_f_* = 0.34 (S_3_); HPLC: *t_R_* = 0.851; MS calcd for [M + H]^+^: C_17_H_13_N_3_O_3_ *m/z*: 307.30, found: 308.29; ^1^H-NMR (300 MHz, CDCl_3_-*d*) δ ppm 3.96 (s, 3 H) 5.14 (s, 2 H) 7.15 (d, *J* = 8.46 Hz, 1 H) 7.18–7.28 (m, 3 H) 7.41 (dd, *J* = 7.18, 0.51 Hz, 1 H) 7.63 (dd, *J* = 8.46, 7.44 Hz, 2 H).

##### 4-Methoxy-2-(2-phenylethyl)-1*H*-isoindole-1,3(2*H*)-dione (**12**)

White powdery crystals. Yield: 43%; mp 123–124 °C; TLC: *R_f_* = 0.73 (S_1_); HPLC: *t_R_* = 1.533; MS calcd for [M + H]^+^: C_17_H_15_NO_3_ *m/z*: 281.31, found: 282.05; ^1^H-NMR (300 MHz, CDCl_3_-*d*) δ ppm 2.93–3.00 (m, 2 H) 3.85–3.92 (m, 2 H) 4.01 (s, 3 H) 7.15–7.32 (m, 6 H) 7.38–7.42 (m, 1 H) 7.63 (dd, *J* = 8.46, 7.44 Hz, 1 H).

##### 4-Methoxy-2-[2-(pyridin-2-yl)ethyl]-1*H*-isoindole-1,3(2*H*)-dione (**13**)

Creamy powdery crystals. Yield: 71%; mp 155–157 °C; TLC: *R_f_* = 0.24 (S_1_); HPLC: *t_R_* = 0.797; MS calcd for [M + H]^+^: C_16_H_14_N_2_O_3_ *m/z*: 282.10, found: 283.11; ^1^H-NMR (300 MHz, CDCl_3_-*d*) δ ppm 3.12–3.19 (m, 2 H) 4.00 (s, 3 H) 4.06 (dd, *J* = 7.95, 6.67 Hz, 2 H) 7.08–7.20 (m, 3 H) 7.39 (dd, *J* = 7.31, 0.64 Hz, 1 H) 7.52–7.67 (m, 2 H) 8.49–8.53 (m, 1 H).

##### 2-[2-(1*H*-Imidazol-4-yl)ethyl]-4-methoxy-1*H*-isoindole-1,3(2*H*)-dione (**14**)

White powdery crystals. Yield: 30%; mp 227–228 °C; TLC: *R_f_* = 0.14 (S_2_); HPLC: *t_R_* = 0.774; MS calcd for [M + H]^+^: C_14_H_13_N_3_O_3_ *m/z*: 271.27, found: 272.14; ^1^H-NMR (300 MHz, DMSO-*d*_6_) δ ppm 2.81–2.88 (m, 2 H) 3.74 (t, *J* = 6.80 Hz, 2 H) 3.92 (s, 3 H) 7.11 (s, 1 H) 7.33–7.47 (m, 2 H) 7.75 (dd, *J* = 8.46, 7.44 Hz, 1 H) 8.24 (d, *J* = 1.03 Hz, 1 H).

##### 2-[2-(1*H*-Benzimidazol-2-yl)ethyl]-4-methoxy-1*H*-isoindole-1,3(2*H*)-dione (**15**)

Creamy powdery crystals. Yield: 40%; mp 197–198 °C; TLC: *R_f_* = 0.37 (S_3_); HPLC: *t_R_* = 0.871; MS calcd for [M + H]^+^: C_18_H_15_N_3_O_3_ *m/z*: 321.11, found: 322.31; ^1^H-NMR (300 MHz, DMSO-*d*_6_) δ ppm 3.10 (t, *J* = 7.33 Hz, 2 H) 3.89–3.98 (m, 5 H) 7.05–7.12 (m, 2 H) 7.37 (d, *J* = 7.62 Hz, 1 H) 7.39–7.47 (m, 3 H) 7.72–7.79 (m, 1 H).

##### 4-Methoxy-2-(3-phenylpropyl)-1*H*-isoindole-1,3(2*H*)-dione (**16**)

White powdery crystals. Yield: 73%; mp 94–95 °C; TLC: *R_f_* = 0.80 (S_1_); HPLC: *t_R_* = 1.572; MS calcd for [M + H]^+^: C_18_H_17_NO_3_ *m/z*: 295.21, found: 296.32; ^1^H-NMR (300 MHz, CDCl_3_-*d*) δ ppm 2.01 (dt, *J* = 14.68, 7.66 Hz, 2 H) 2.62–2.71 (m, 2 H) 3.71 (t, *J* = 7.18 Hz, 2 H) 4.01 (s, 3 H) 7.10–7.28 (m, 6 H) 7.41 (dd, *J* = 7.18, 0.51 Hz, 1 H) 7.64 (dd, *J* = 8.34, 7.31 Hz, 1 H).

##### 2-[3-(1*H*-Benzimidazol-2-yl)propyl]-4-methoxy-1*H*-isoindole-1,3(2*H*)-dione (**17**)

Creamy powdery crystals. Yield: 45%; mp 217–218 °C; TLC: *R_f_* = 0.37 (S_2_); HPLC: *t_R_* = 0.910; MS calcd for [M + H]^+^: C_19_H_17_N_3_O_3_ *m/z*: 335.13, found: 336.34; ^1^H-NMR (300 MHz, CDCl_3_-*d*) δ ppm 2.12–2.22 (m, 2 H) 2.88–2.96 (m, 2 H) 3.71–3.80 (m, 2 H) 4.06 (s, 3 H) 7.18–7.28 (m, 4 H) 7.47 (dd, *J* = 7.31, 0.64 Hz, 1 H) 7.59 (dd, *J* = 5.51, 2.95 Hz, 1 H) 7.70 (dd, *J* = 8.46, 7.44 Hz, 1 H).

##### 2-[4-(1*H*-Benzimidazol-2-yl)butyl]-4-methoxy-1*H*-isoindole-1,3(2*H*)-dione (**18**)

Creamy powdery crystals. Yield: 45%; mp 69–70 °C; TLC: *R_f_* = 0.28 (S_2_); HPLC: *t_R_* = 1.002; C_20_H_19_N_3_O_3_ *m/z*: 349.14, found: 350.17; ^1^H-NMR (300 MHz, CDCl_3_-*d*) δ ppm 1.67–1.92 (m, 4 H) 2.97 (t, *J* = 7.33 Hz, 2 H) 3.67 (t, *J* = 6.74 Hz, 2 H) 3.95 (s, 3 H) 7.12–7.20 (m, 3 H) 7.37 (d, *J* = 7.03 Hz, 1 H) 7. –7.55 (m, 2 H) 7.61 (t, *J* = 7.91 Hz, 1 H); ^13^C-NMR (75 MHz, CDCl_3_-*d*) δ ppm 25.26, 27.83, 28.27, 36.80, 56.28, 115.46, 117.15, 117.52, 122.08, 134.09, 136.21, 154.51, 156.59, 167.36, 168.25.

##### 2-[5-(1*H*-Benzimidazol-2-yl)pentyl]-4-methoxy-1*H*-isoindole-1,3(2*H*)-dione (**19**)

Creamy powdery crystals. Yield: 50%; mp 108–109 °C; TLC: *R_f_* = 0.38 (S_2_); HPLC: *t_R_* = 1.072; MS calcd for [M + H]^+^: C_21_H_21_N_3_O_3_ *m/z*: 363.41, found: 364.12; ^1^H-NMR (300 MHz, CDCl_3_-*d*) δ ppm 1.32–1.46 (m, 2 H) 1.69 (quin, *J* = 7.18 Hz, 2 H) 1.90 (dt, *J* = 15.68, 7.69 Hz, 2 H) 2.91 (t, *J* = 7.33 Hz, 2 H) 3.64 (t, *J* = 7.03 Hz, 2 H) 4.00 (s, 3 H) 7.14–7.21 (m, 3 H) 7.41 (d, *J* = 7.03 Hz, 1 H) 7.53 (dd, *J* = 5.86, 2.93 Hz, 2 H) 7.64 (dd, *J* = 8.50, 7.33 Hz, 1 H); ^13^C-NMR (75 MHz, CDCl_3_-*d*) δ ppm 26.07, 27.13, 28.03, 29.00, 37.21, 56.28, 115.43, 117.24, 117.47, 122.02, 134.17, 136.15, 154.73, 156.57, 167.35, 168.23.

##### 2-[2-(1*H*-Benzimidazol-2-yl)ethyl]-1*H*-isoindole-1,3(2*H*)-dione (**20**)

White powdery crystals. Yield: 55%; mp 202–203 °C; TLC: *R_f_* = 0.35 (S_3_); HPLC: *t*_R_ = 0.827; MS calcd for [M + H]^+^: C_17_H_13_N_3_O_2_ *m/z*: 291.30, found: 292.06; ^1^H-NMR (300 MHz, DMSO-*d*_6_) δ ppm 3.14 (t, *J* = 7.33 Hz, 2 H) 4.00 (t, *J* = 7.33 Hz, 2 H) 7.06–7.12 (m, 2 H) 7.38–7.46 (m, 2 H) 7.78–7.87 (m, 4 H).

##### 2-[3-(1*H*-Benzimidazol-2-yl)propyl]-1*H*-isoindole-1,3(2*H*)-dione (**21**)

White powdery crystals. Yield: 66%; mp 190–191 °C; TLC: *R_f_* = 0.37 (S_3_); HPLC: *t_R_* = 0.869; MS calcd for [M + H]^+^: C_18_H_15_N_3_O_2_ *m/z*: 305.12, found: 306.10; ^1^H-NMR (300 MHz, CDCl_3_-*d*) δ ppm 2.15–2.27 (m, 2 H) 2.89–2.98 (m, 2 H) 3.74–3.83 (m, 2 H) 7.17–7.25 (m, 2 H) 7.53–7.61 (m, 2 H) 7.71–7.78 (m, 2 H) 7.82–7.89 (m, 2 H).

##### 2-[4-(1*H*-Benzimidazol-2-yl)butyl]-1*H*-isoindole-1,3(2*H*)-dione (**22**)

Creamy powdery crystals. Yield: 43%; mp 179–180 °C; TLC: *R_f_* = 0.28 (S_2_); HPLC: *t_R_* = 1.001; MS calcd for [M + H]^+^: C_19_H_17_N_3_O_2_ *m/z*: 319.13, found: 320.10; ^1^H-NMR (300 MHz, CDCl_3_-*d*) δ ppm 1.73–1.94 (m, 4 H) 3.00 (t, *J* = 7.33 Hz, 2 H) 3.75 (t, *J* = 6.74 Hz, 2 H) 7.16–7.23 (m, 2 H) 7.49–7.58 (m, 2 H) 7. –7.74 (m, 2 H) 7.79–7.86 (m, 2 H).

##### 2-[5-(1*H*-Benzimidazol-2-yl)pentyl]-1*H*-isoindole-1,3(2*H*)-dione (**23**)

White powdery crystals. Yield: 59%; mp 168–169 °C; TLC: *R_f_* = 0.30 (S_2_); HPLC: *t_R_* = 1.080; MS calcd for [M + H]^+^: C_20_H_19_N_3_O_2_ *m/z*: 333.15, found: 334.15; ^1^H-NMR (300 MHz, CDCl_3_-*d*) δ ppm 1.37–1.50 (m, 2 H) 1.74 (quin, *J* = 7.18 Hz, 2 H) 1.94 (dt, *J* = 15.39, 7.84 Hz, 2 H) 2.93 (t, *J* = 7.33 Hz, 2 H) 3.70 (t, *J* = 7.03 Hz, 2 H) 7.16–7.23 (m, 2 H) 7.54 (dd, *J* = 5.86, 2.93 Hz, 2 H) 7.67–7.74 (m, 2 H) 7.79–7.86 (m, 2 H).

### 3.2. Pharmacology

#### 3.2.1. Protocols for Measuring PDE10A Inhibition In Vitro

Test and reference compounds were dissolved in dimethyl sulfoxide (DMSO) at a concentration of 1 mM and further diluted in assay buffer in order to obtain the final concentration of compounds: 10 µM and 3 µM, respectively. All reactions were carried out at 37 °C in white, half-area 96-well plates (Perkin Elmer, Waltham, MA, USA). The inhibition of PDE 10A enzyme was measured using the PDElight HTS cAMP phosphodiesterase assay kit (Lonza) according to the manufacturer’s recommendations. 2.5 U of PDE 10A enzyme was preincubated either with DMSO (vehicle control) or compound for 20 min before incubation with the substrate, cAMP (final concentration 1.25 µM), for 1 h. Then, PDELight AMP Detection Reagent was added. After 10 min incubation, the luminescence was measured in a multifunction plate reader (POLARstar Omega, BMG Labtech, Ortenberg, Germany). The results were expressed as percent of inhibition or, for the most active compounds selected, as the half maximal inhibitory concentration values.

#### 3.2.2. Radioligand Binding Studies

Radioligand binding studies with serotonin 5-HT_1A_ and 5-HT_7_ receptors were conducted according to methods previously described [41,42], whereas percent of inhibition of the control binding for 5-HT_2A_ and D_2_ were performed according to protocols described online (www.cerep.fr). Briefly: binding experiments were conducted in 96-well microplates in a total final volume of 250 μL of appropriate buffers. Reaction mix included 50 μL solution of test compound, 50 μL of radioligand ([^3^H]8-OH-DPAT for 5-HT_1A_, and [^3^H]-LSD for 5-HT_7_, respectively) and 150 μL of diluted membranes (see description and Table S2 in Supporting Materials). Specific assay conditions for each receptor are described elsewhere [41]. The radioactivity was measured in a MicroBeta2 scintillation counter (PerkinElmer, USA). Each compound was tested in the assay as duplicate samples at 1 µM final concentrations. Results were expressed as percent inhibition of specific binding.

### 3.3. Molecular Modeling

The phosphodiesterase 10A model was developed on the basis of the experimental structure of the enzyme (PDB ID: 3SNI) [43]. The structure was refined using default settings in the Protein Preparation Wizard. Water molecules (except for the buried water molecules) and hetero groups, other than the ligand, were deleted and the whole system was minimized (OPLS3 force field). The model was tested extensively with docking studies involving PDE 10A inhibitors. The resulting consistent binding modes of the reference compounds of experimentally proven affinity were used as the basis for verifying the accuracy of the model that served as molecular target in docking studies. Ligand structures were optimized using the LigPrep tool. The Glide SP flexible docking procedure was carried out using default parameters. H-bond constraint, as well as centroid of a grid box, were located on Gln716.

Glide, LigPrep, and Protein Preparation Wizard were implemented in the Small-Molecule Drug Discovery Suite (Schrödinger, Inc., NY, USA), which was licensed to the Jagiellonian University Medical College.

### 3.4. Cytotoxicity

#### 3.4.1. Cells Culture

The HePG2 human liver cancer cell-line, (ATCC^®^ 59195™) and the SH-SY5Y human neuroblastoma cell-line (ATCC^®^ CRL-2266™) were used in the study. The cells were cultured in standard conditions (37 °C, 5% CO_2_), in EMEM (HepG2 and SH-SY5Y), supplemented with 10% fetal bovine serum (FBS) (Gibco, Thermo Fisher Scientific, Waltham, MA. USA) and antibiotics (Lonza, Köln, Germany).

#### 3.4.2. Cytotoxicity Analysis—MTT

The MTT assay was used to determine the cytotoxic effects of the analyzed compounds. Cells were seeded at a density of 2 × 10^4^ in 96-well plates. Following overnight culture, cells were then treated with increasing concentrations of compound **18** (0.1–100 µM) and incubated for 24 h. Following cell exposure for 24 h, 10 µL MTT reagent (Sigma Aldrich) was added to each well and, after 3 h of incubation (37 °C, 5% CO_2_), the medium was aspirated and the formazan produced in the cells appeared as dark crystals in the bottom of the wells. Next, Crystal DMSO was added to each well. Then, the optical density (OD) of each well was determined at 570 nm on a plate-reader (Spectra iD3, Molecular Devices, San Jose, CA, USA). The number of metabolically active and living cells is directly proportional to the absorbance of the samples. The results are presented in Figure 5 as the percentage of control ± SEM. Doxorubicin was used as reference standard (cytotoxic agent).

### 3.5. In Vivo Studies

The experiments were performed on male CD-1 mice (in the accredited animal facility at the Jagiellonian University Medical College, Krakow, Poland), and mice were kept in groups of ten in Makrolon type 3 cages (dimensions 26.5 × 15 × 42 cm). The animals were kept in an environmentally controlled room (ambient temperature 22 ± 2 °C; relative humidity 50–60%; 12:12 light:dark cycle, lights on at 8:00). They were allowed to acclimatize with the environment for one week before commencement of the experiments. Standard laboratory food (Ssniff M-Z) and filtered water were freely available. All the experimental procedures were approved by the I Local Ethics Commission at the Jagiellonian University in Krakow (Approval Nos.: 125/2017, 123/2015, and 158/2017).

All the experiments were conducted in the light phase between 09.00 and 14.00 h. Each experimental group consisted of 7–10 animals per treatment dose. The animals were used only once.

#### 3.5.1. *d*-Amphetamine-Induced Hyperlocomotor Activity in CD-1 Mice

Locomotor activity was recorded with an Opto M3 multi-channel activity monitor (MultiDevice Software v.1.3, Columbus Instruments, Columbus, OH, USA). The CD-1 mice were individually placed in plastic cages (22 × 12 × 13 cm) immediately after drug administration, and then ambulation was counted during 1 h with data recording every 10 min. The cages were cleaned up with 70% ethanol after each mouse.

#### 3.5.2. Spontaneous Locomotor Activity in CD-1 Mice

Locomotor activity was recorded according to the method described above.

#### 3.5.3. Drugs

The following drugs were used: d-amphetamine (sulfate, Sigma-Aldrich, Poznań, Poland), papaverine (hydrochloride, Sigma-Aldrich, Poznań, Poland), and tested compound **18**. D-amphetamine and papaverine were dissolved in distilled water; the tested compound (**18**) was suspended in a 1% aqueous solution of Tween 80 immediately before administration. Papaverine and **18** were administered intraperitoneally (ip) and d-amphetamine subcutaneously (sc) just prior to the test starting. All compounds were injected at a volume of 10 mL/kg. Control animals received a vehicle injection according to the same schedule. Figure 6 shows only the doses at which the antipsychotic-like activity of AMPH, papaverine, and compound **18** were observed.

#### 3.5.4. Statistics

All the data are presented as the mean ± SEM. The statistical significance of the results was evaluated by a one-way ANOVA, followed by a Bonferroni’s Comparison Test.

## 4. Conclusions

In this study, we describe the synthesis of a library of novel 4-methoxy-2,3-dihydro-1*H*-isoindole-1,3-dione derivatives with various aminoalkyl moieties, as potential inhibitors of PDE10A and serotonin receptor ligands. Among the synthesized and tested compounds, 1*H*-benzimidazole derivatives were identified as potent PDE10A inhibitors. Compound **18**, the most potent PDE10 inhibitor (IC_50_ = 886 ± 0.017 nM) of the group, with drug-like properties as defined by Lipinski’s Rule of five, was selected for further pharmacological evaluation. In order to explain its inhibitory activity towards PDE10A, the binding mode of compound **18** was determined. Molecular modeling studies showed that H-bonds formed by the 4-methoxy group in the phthalimide moiety as well as the optimal length of the carbon linker are crucial for interactions with PDE10A. Preliminary in vitro neurotoxicity and hepatotoxicity studies revealed that the compound is safe and did not exhibit noteworthy cytotoxicity. Furthermore, significant antipsychotic properties of 2-[4-(1*H*-benzimidazol-2-yl)butyl]-4-methoxy-1*H*-isoindole-1,3(2*H*)-dione (compound **18**) was identified in the d-AMPH-induced hyperlocomotor activity test in mice, but further pharmacological studies are needed to explain the mechanism underlying its antipsychotic activity. This study successfully identified a number of compounds with significant PDE10A inhibitory activity and provided further information into the molecular basis of their interaction with PDE10A.

## Figures and Tables

**Table 1:** Screening data from a luminescence PDE inhibition assay and a radioligand binding assay for 5-HT_1A_ and 5-HT_7_ (%) of 4-methoxy-1*H*-isoindole-1,3(2*H*)-dione and 1*H*-isoindole-1,3(2*H*)-dione derivatives displaying phosphodiesterase 10A inhibitory activity and binding affinity for serotonin 5-HT_1A_ and 5-HT_7_ receptors as well as selected IC_50_ values of the most active compounds for PDE10A.

	Compd	R	n	PDE10A [%] ^1^		IC_50_ PDE10A [µM] ± SEM ^3^	5-HT_1A_	5-HT_7_
				**10** **µ** **M**	**3** **µ** **M**		1 µM ^2^ ± SEM ^3^	
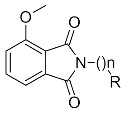	**3**	A	2	0	0	-	27 ± 3	0 ± 0
	**4**	B	2	0	0	-	12 ± 4	0 ± 0
	**5**	C	2	0	0	-	25 ± 3	0 ± 0
	**6**	A	3	0	0	-	32 ± 4	0 ± 0
	**7**	B	3	19	18	-	33 ± 2	51 ± 2
	**8**	C	3	7	7	-	28 ± 3	0 ± 0
	**9**	D	1	0	0	-	6 ± 2	0 ± 0
	**10**	E	1	0	0	-	10 ± 1	0 ± 2
	**11**	F	1	15	7	-	39 ± 3	0 ± 1
	**12**	D	2	10	5	-	13 ± 2	0 ± 0
	**13**	E	2	0	0	-	59 ± 4	0 ± 2
	**14**	G	2	0	0	-	21 ± 1	0 ± 0
	**15**	F	2	70	40	6.710 ± 0.195	44 ± 5	0 ± 3
	**16**	D	3	13	11	-	32 ± 3	14 ± 0
	**17**	F	3	86	73	1.001 ± 0.009	16 ± 5	10 ± 3
	**18 ^4^**	F	4	92	79	0.886 ± 0.017	28 ± 3	3 ± 1
	**19**	F	5	75	52	4.305 ± 0.365	28 ± 2	6 ± 0
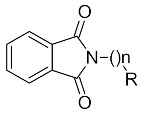	**20**	F	2	1	0	-	26 ± 2	36 ± 0
	**21**	F	3	45	16	-	27 ± 2	14 ± 2
	**22**	F	4	73	47	5.645 ± 0.495	20 ± 3	15 ± 2
	**23**	F	5	58	26	-	48 ± 3	34 ± 2
Papaverine				75	54	5.755 ± 0.055	-	-
Methiothiepin				-	-	-	100 ± 0	99 ± 1
DMSO				0	0	-	-	-

^1^ The percentage of inhibition was calculated respective to vehicle control (DMSO); A: 6,7-dimethoxy-1,2,3,4-tetrahydroisoquinoline, B: 1,2,3,4-tetrahydroisoquinoline, C: decahydroisoquinoline, D: phenyl, E: 2-pyridine, F: 2-benzimidazole, G: 1*H*-imidazole; ^2^ % of control specific binding; ^3^ SEM of three independent experiments performed in duplicate; ^4^ Additionally, the percent of inhibition of the control binding for 5-HT_2A_ and D_2_ for compound **18** were evaluated. The screening results were 8.8% and 0%, for 5-HT_2A_ and for D_2_ receptors, respectively, at a concentration of 1 µM. The screening tests for 5-HT_2A_ and D_2_ receptors were performed in the CEREP Laboratories, France—Eurofins Scientific (www.cerep.fr).

## Supplementary Materials

The following are available online, Figure S1: The spider graph of drug-like parameters of compound **18**, Figure S2. Effects of papaverine and compound **18** on d-amphetamine-induced hyperlocomotor activity in CD-1 mice, Table S1: Effects of papaverine and compound **18** on spontaneous locomotor activity in CD-1 mice, Table S2. Detailed conditions of the in vitro binding assays for the 5-HT_1A_ and 5-HT_7_ receptors and examples of NMR spectra.
