# Supplementary material for: Impact of N-Alkylamino Substituents on Serotonin Receptor (5-HTR) Affinity and Phosphodiesterase 10A (PDE10A) Inhibition of Isoindole-1,3-dione Derivatives
Source: Molecules. 2020 Aug 25;25(17):3868. doi: 10.3390/molecules25173868 (PMC7504677; doi:10.3390/molecules25173868)
Supplement: Supplementary file 1 [file molecules-25-03868-s001.pdf]

Supplementary Materials:

Impact of N-Alkylamino Substituents on Serotonin Receptor (5-HTR) Affinity and Phosphodiesterase 10A (PDE10A) Inhibition of Isoindole-1,3-dione Derivatives

Anna Czopek [1,*], Anna Partyka [2], Adam Bucki [1], Maciej Pawłowski [1], Marcin Kołaczkowski [1], Agata Siwek [3], Monika Głuch-Lutwin [3], Paulina Koczurkiewicz [4], Elżbieta Pękala [4], Anna Jaromin [5], Bożena Tyliszczak [6], Anna Wesołowska [2] and Agnieszka Zagórska [1]

[1] Department of Medicinal Chemistry, Jagiellonian University Medical College, 9 Medyczna Street, 30-688 Krakow, Poland; adam.bucki@uj.edu.pl (A.B.); maciej.pawlowski@uj.edu.pl (M.P.); marcin.kolaczkowski@uj.edu.pl (M.K.); agnieszka.zagorska@uj.edu.pl (A.Z.)

[2] Department of Clinical Pharmacy, Jagiellonian University Medical College, 9 Medyczna Street, 30-688 Krakow, Poland; annairena.partyka@uj.edu.pl (A.P.); a.wesolowska@uj.edu.pl (A.W.)

[3] Department of Pharmacobiology, Jagiellonian University Collegium Medicum, 9 Medyczna Street, 30-688 Krakow, Poland; agat.siwek@uj.edu.pl (A.S.); monika.gluch-lutwin@uj.edu.pl (M.G.-L.)

[4] Department of Pharmaceutical Biochemistry, Jagiellonian University Collegium Medicum, 9 Medyczna Street, 30-688 Krakow, Poland; paulina.koczurkiewicz@uj.edu.pl (P.K.); elzbieta.pekala@uj.edu.pl (E.P.)

[5] Department of Lipids and Liposomes, Faculty of Biotechnology, Uniwersity of Wroclaw, 14a Joliot-Curie, 50-383 Wroclaw, Poland; anna.jaromin@uwr.edu.pl

[6] Faculty of Materials Engineering and Physics, Cracow University of Technology, Institute of Materials Science, 24 Warszawska Street, 31-155 Krakow, Poland; bozena.tyliszczak@pk.edu.pl

* Correspondence: anna.czopek@uj.edu.pl; Tel.: +48-12-6205450

Academic Editor: Josef Jampilek
Received: 4 August 2020; Accepted: 23 August 2020; Published: date

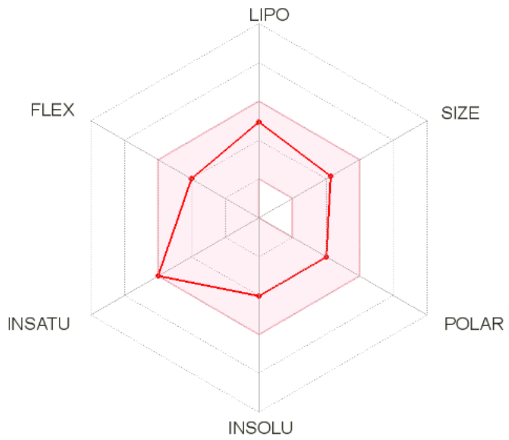

Figure 1. A spider graph of drug-like parameters of compound **18** (source: swissadme website: http://www.swissadme.ch/).

Supplementary materials for *in vivo* pharmacology

Table 1. Effects of papaverine and compound **18** on spontaneous locomotor activity in CD-1 mice.

	Minutes of the test	Vehicle			Papaverine (40 mg/kg)			Papaverine (60 mg/kg)			Cmpd 18 (60 mg/kg)		
Number of movements	**10**	**1157.2**	±	**81.1**	**43.1**	±	**16.6[d]**	7.1	±	5.2[d]	176.2	±	28.8[d]
	20	1849.6	±	153.7	170.2	±	63.8[d]	24.6	±	14.6[d]	285.0	±	77.4[d]
	30	2448.3	±	236.7	571.9	±	128.2[d]	47.5	±	31.0[d]	659.6	±	193.0[c]
	60	3666.7	±	354.2	2260.8	±	306.6[a]	200.4	±	172.1[d]	1875.4	±	474.0[a]

Data are expressed as the mean ± S.E.M. [a] $p<0.05$, [c] $p<0.001$, [d] $p<0.0001$ versus respective vehicle-treated group (one-way ANOVA followed by Bonferroni's *post-hoc* test).

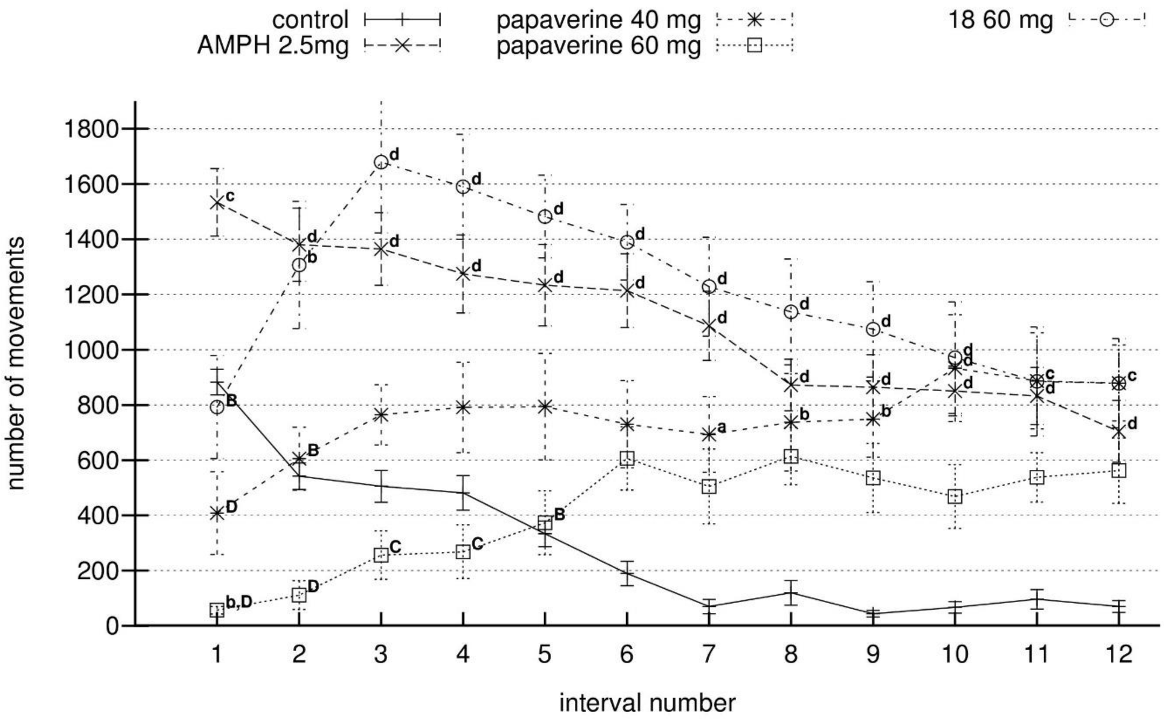

Figure 2. Effects of papaverine and compound 18 on *d*-amphetamine-induced hyperlocomotor activity in CD-1 mice. Data are expressed as the mean of the number of movements measured in 10-min intervals of two papaverine-treated groups (at doses of 40 and 60 mg, respectively) and compound **18**-treated group (at a dose of 60 mg) ± S.E.M. [a] $p<0.05$, [b] $p<0.01$, [c] $p<0.001$, [d] $p<0.0001$ *versus* respective control vehicle-treated group, [B] $p<0.01$, [C] $p<0.001$, [D] $p<0.0001$ and respective AMPH-treated group (one-way ANOVA followed by Bonferroni's *post-hoc* test). AMPH – *d*-amphetamine.

Examples of NMR spectra

Compound 4

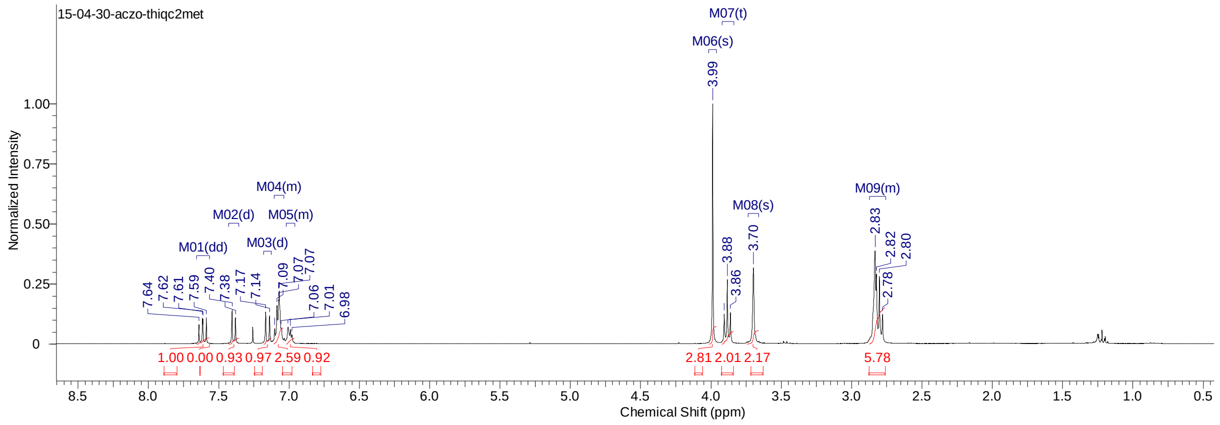

Compound 6

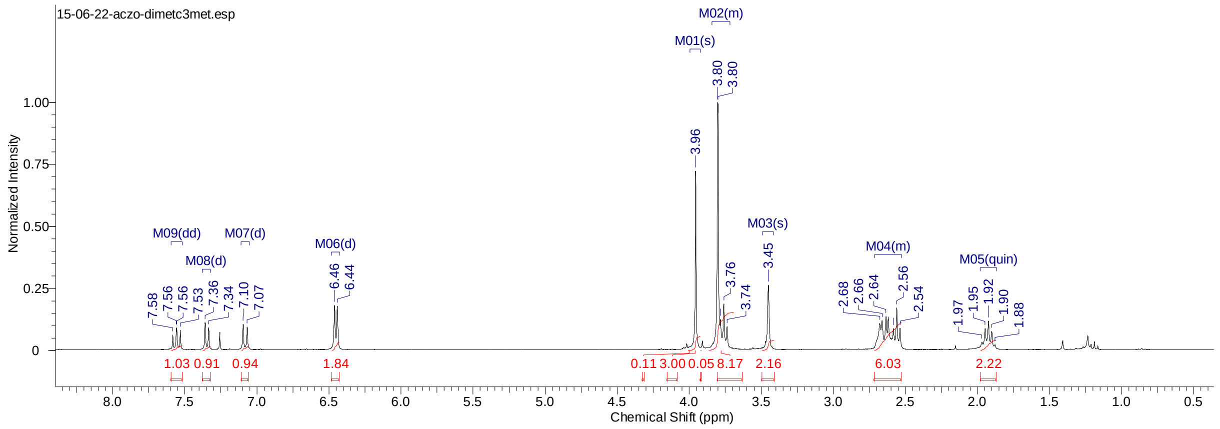

Compound 8

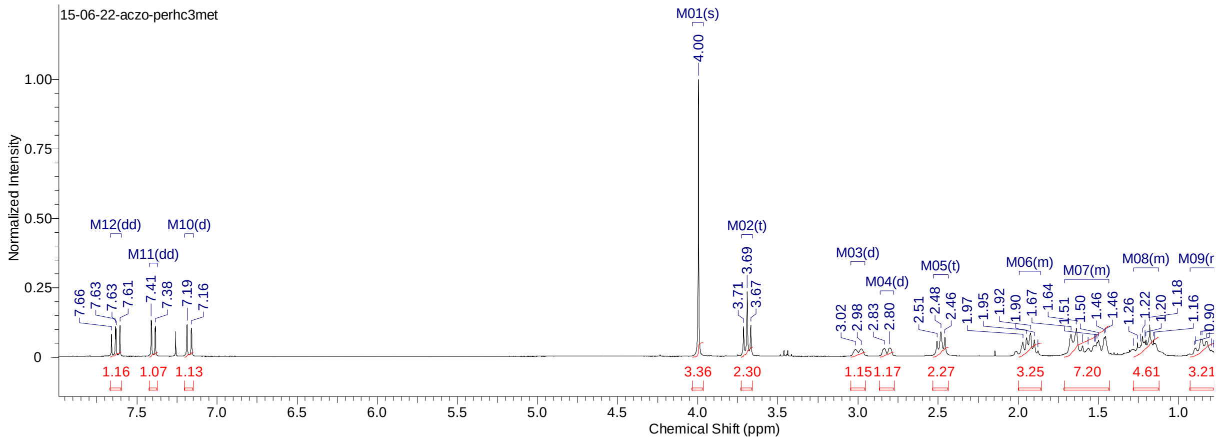

Compound 12

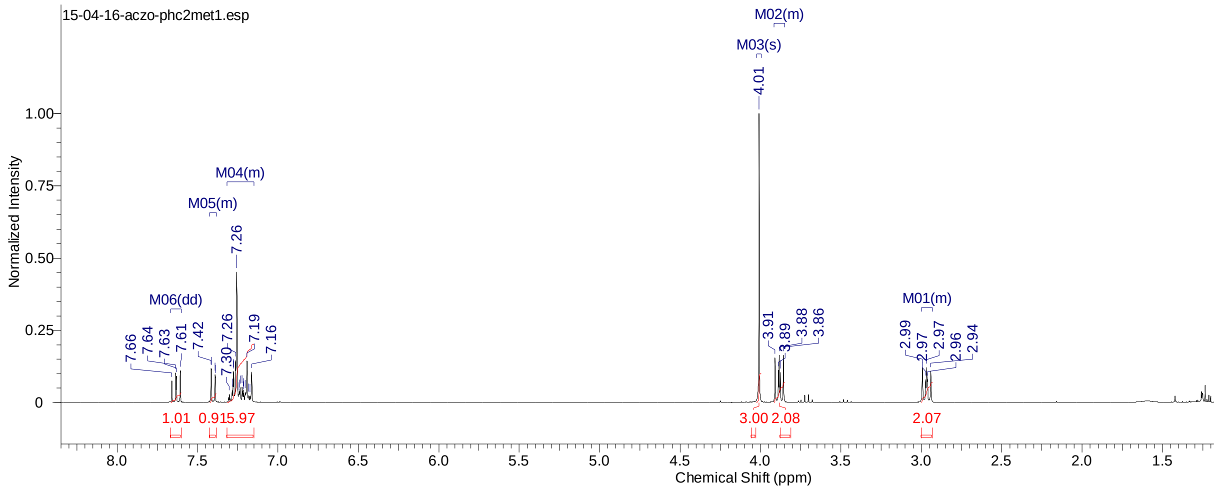

Compound 13

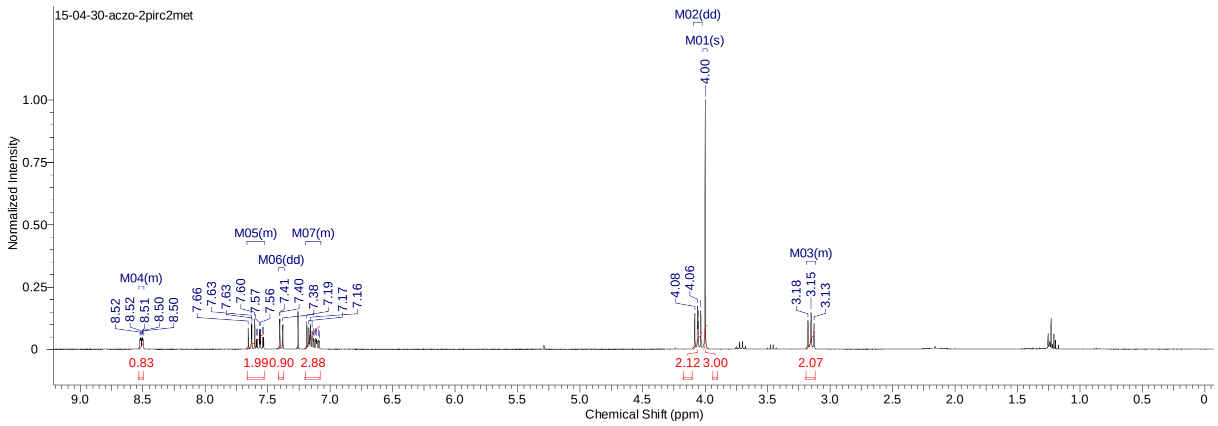

Compound 15

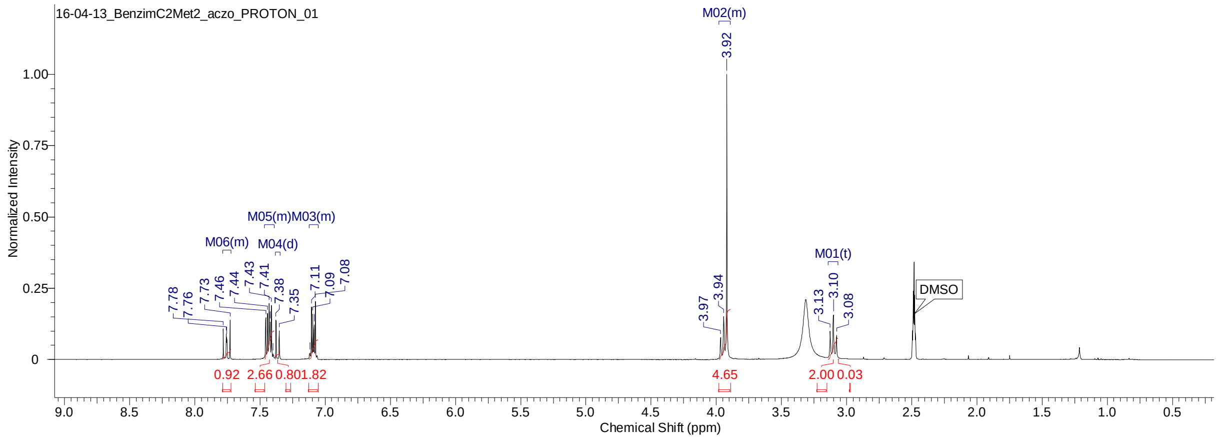

Compound 17

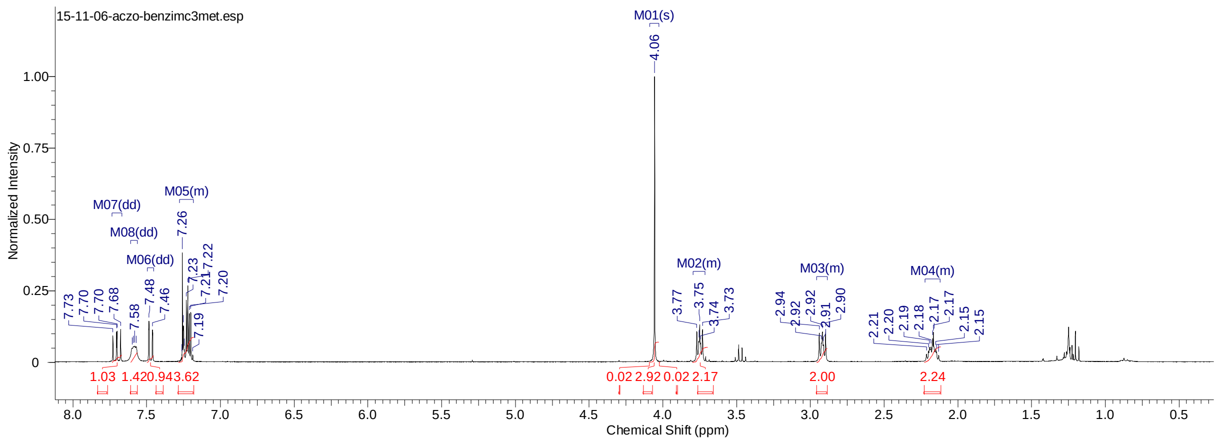

Compound 18 (H NMR)

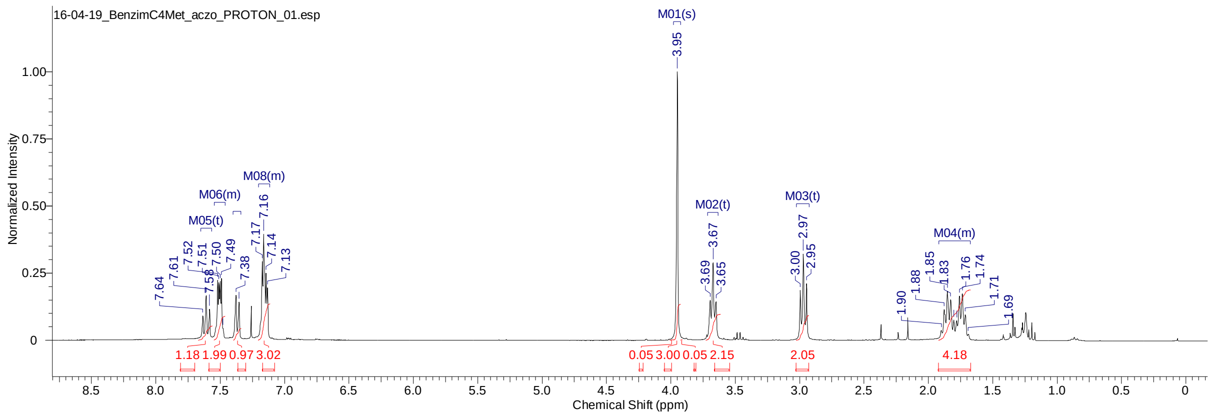

Compound 18 (C NMR)

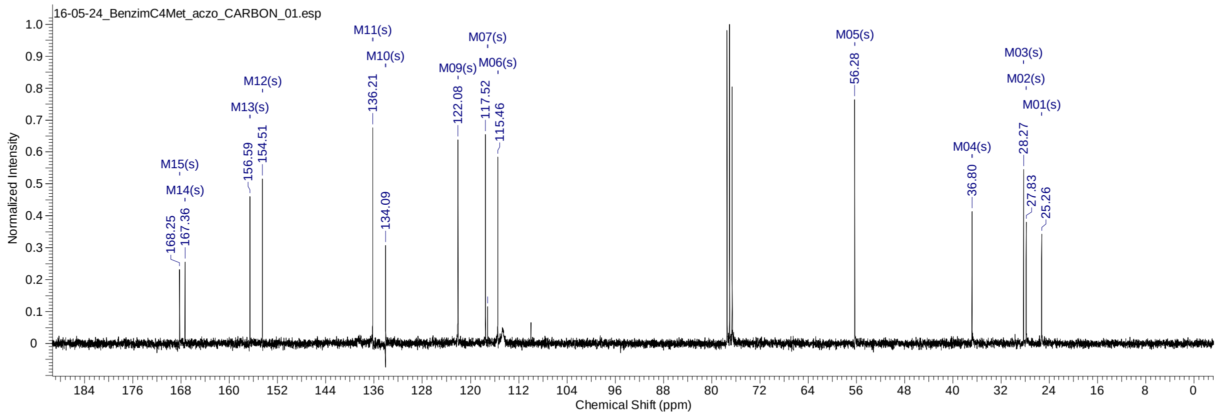

Compound 19 (H NMR)

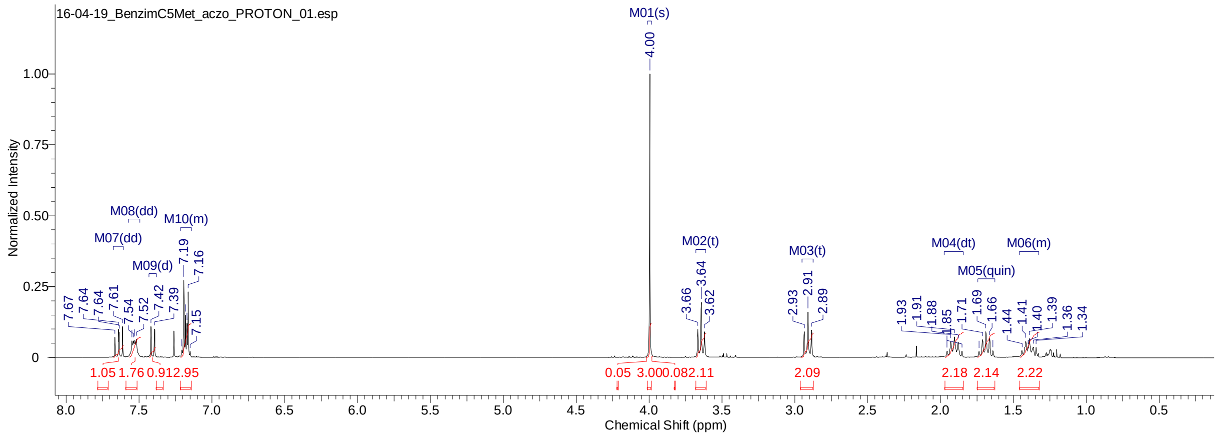

Compound 19 (C NMR)

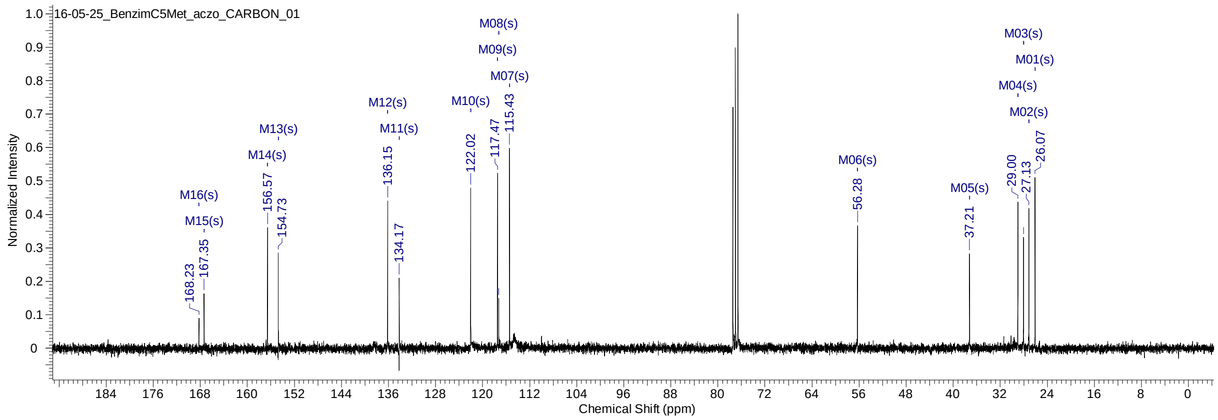

Compound 21

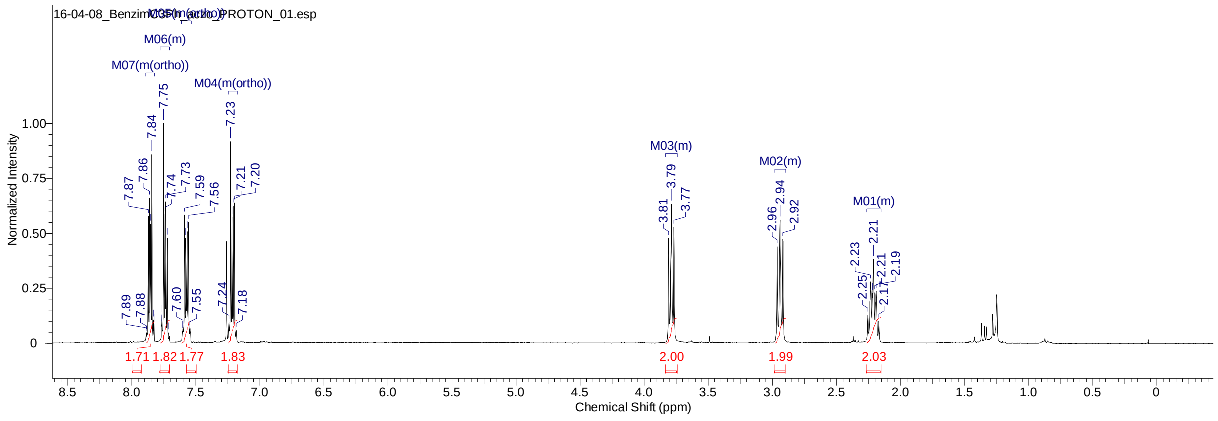

Compound 23

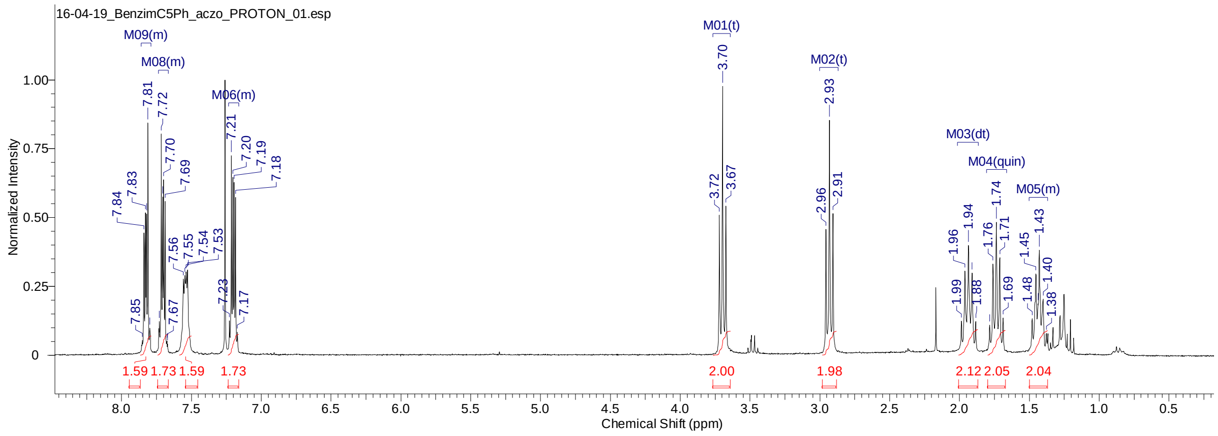
16-04-19_BenzimC5Ph_aczo_PROTON_01.esp
M09(m)
M08(m)
M06(m)
M01(t)
M02(t)
M03(dt)
M04(quin)
M05(m)
Normalized Intensity
Chemical Shift (ppm)
1.59 1.73 1.59 1.73 2.00 1.98 2.12 2.05 2.04
